# Exercise-based interventions for sarcopenic obesity in middle-aged and older adults: an umbrella review of systematic reviews with pairwise meta-analyses and network meta-analyses

**DOI:** 10.3389/fnut.2026.1859967

**Published:** 2026-06-10

**Authors:** Xiaoge Ma, Guanping Sun, Chuannan Liu, Xujie Yan, Weibao Liang, Zhengda Ma

**Affiliations:** 1 School of Physical Education and Leisure, Guangdong Ocean University, Zhanjiang, China; 2 Department of Physical Education, Kunsan National University, Gunsan-si, Republic of Korea; 3 School of Physical Education and Health, Zunyi Medical University, Zunyi, China; 4 School of Physical Education and Sports Science, Hengyang Normal University, Hengyang, China; 5 Faculty of Health Sciences and Sports, Macao Polytechnic University, Macao, Macao SAR, China; 6 Hebei Sport University, Shijiazhuang, Hebei, China

**Keywords:** AMSTAR-2, body composition, corrected covered area, exercise, physical function, resistance training, sarcopenic obesity, umbrella review

## Abstract

**Introduction:**

Sarcopenic obesity (SO) is an increasingly important nutrition- and exercise-relevant phenotype in middle-aged and older adults, but the secondary evidence base is complicated by heterogeneous SO definitions, mixed exercise and nutrition interventions, and repeated use of the same primary trials across reviews.

**Methods:**

We conducted an umbrella review of systematic reviews with pairwise meta-analysis or network meta-analysis. PubMed, Web of Science, SPORTDiscus, Cochrane Library, Embase and Scopus were searched from inception to 1 March 2026. Eligible reviews focused on middle-aged and/or older adults with SO and reported quantitative syntheses for body composition and/or physical function. Broader non-pharmacological reviews were retained only when exercise-related quantitative syntheses were separately reported and extractable. Two reviewers independently screened records, extracted review-level data and assessed methodological quality with AMSTAR-2; disagreements were resolved by discussion and, where necessary, third-reviewer adjudication. Primary-study overlap was quantified using the corrected covered area (CCA).

**Results:**

Eight reviews met strict exercise-based eligibility criteria, including seven pairwise meta-analyses and one network meta-analysis. They represented 33 unique primary-study units and 88 review-level primary-study occurrences. Primary-study overlap was very high (CCA = 0.238). AMSTAR-2 confidence was low in seven reviews and critically low in one, and umbrella-level outcome certainty was judged low or very low. Adiposity outcomes were directionally favorable across all eight reviews, and physical-function outcomes were generally favorable, particularly for grip strength and gait- or mobility-related performance. Muscle-mass outcomes were mixed, while metabolic and inflammatory outcomes were sparse and inconsistent. Resistance training showed the most consistent strength-related signal, whereas combined or multicomponent exercise provided hypothesis-supporting, not definitive, signals when adiposity and mobility targets were considered together.

**Discussion:**

Exercise-centered interventions may be associated with improvements in adiposity and functional outcomes in middle-aged and older adults with SO; however, clinical, modality-ranking and comparative-effectiveness claims should remain cautious because the review corpus is methodologically weak and highly overlapping.

**Systematic review registration:**

PROSPERO CRD420261370155, https://www.crd.york.ac.uk/PROSPERO/view/CRD420261370155

## Graphical abstract

**Figure:**
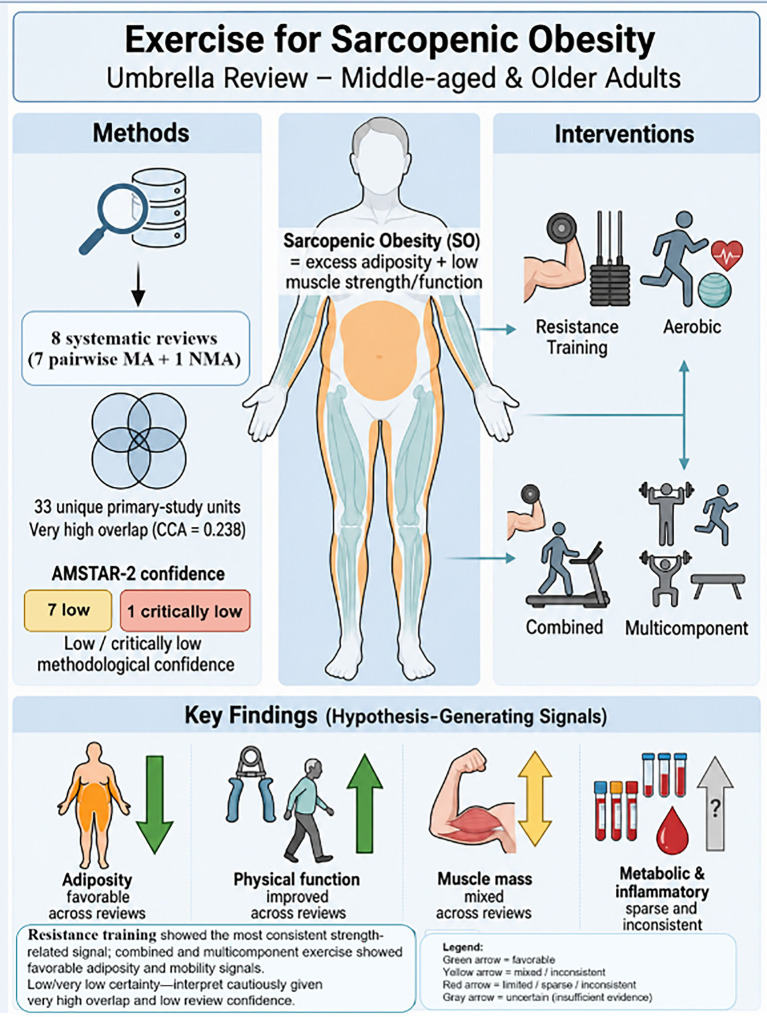
Created with Biorender.com.

## Introduction

Sarcopenic obesity (SO) combines excess adiposity with low muscle mass, low muscle strength and/or impaired physical performance, creating a phenotype that is clinically more complex than obesity or sarcopenia alone. Consensus statements have emphasized that SO should be understood as a disorder of body composition and function rather than as a simple coexistence of two diagnoses (1, 2). Its relevance to nutrition and exercise science is direct: energy balance, protein adequacy, inflammation, insulin resistance, muscle quality and physical activity all contribute to the same risk pathway.

The public-health importance of SO has increased as populations age and obesity remains common in later life. A global meta-analysis estimated that SO affects a meaningful proportion of older adults, although prevalence varies substantially by diagnostic criteria and measurement method (3). SO has been associated with mobility limitation, cardiometabolic complications, disability, lower quality of life and mortality risk, and these associations appear stronger when obesity-related metabolic burden and sarcopenia-related functional impairment coexist (4, 5). An umbrella review of health outcomes has also highlighted that SO is not only a body-composition label but a multi-domain risk state across physical, metabolic and survival outcomes (6).

Operational heterogeneity remains a central obstacle for intervention research. Sarcopenia definitions have evolved through European and Asian consensus updates that place greater emphasis on muscle strength and physical performance, while the ESPEN and EASO consensus and the Sarcopenic Obesity Global Leadership Initiative have attempted to standardize SO diagnosis and research priorities (2, 7–9). Nevertheless, published exercise trials and reviews continue to vary in age thresholds, body-fat indicators, appendicular skeletal muscle indices, grip-strength thresholds and functional cut-points. This heterogeneity makes direct comparison of exercise modalities difficult and increases the risk that apparently consistent review-level findings may reflect repeated synthesis of the same primary trials rather than independent confirmation.

Exercise-centered strategies are biologically plausible and clinically attractive because they can address adiposity, muscle strength, muscle quality, cardiometabolic risk and mobility simultaneously. Resistance exercise is particularly relevant to neuromuscular function and strength preservation, while aerobic, combined and multicomponent programs may be better aligned with adiposity reduction and whole-person mobility goals. Nutritional supplementation may also be used as an adjunct to exercise in SO management, but the present review focuses on exercise-based evidence and treats nutrition-only estimates as outside the primary analytic boundary (4, 10).

The review literature on SO is now crowded with systematic reviews, pairwise meta-analyses and network meta-analyses, including reviews of broad non-pharmacological interventions, exercise-modality comparisons, resistance-training-only syntheses and exercise plus nutrition interventions. Clinicians and nutrition-exercise researchers therefore need an umbrella-level synthesis that separates exercise-based conclusions from broader intervention mixes, appraises methodological confidence and quantifies primary-study overlap. We conducted this umbrella review to summarize published review-level effects of exercise-based interventions for SO in middle-aged and older adults; to distinguish evidence for adiposity, muscle mass and physical function; to describe secondary metabolic and inflammatory outcomes; to appraise review quality; and to quantify overlap across the included review corpus.

A previous umbrella review synthesized nutritional and exercise interventions for SO and used AMSTAR and GRADE, but it included only four meta-analyses and did not quantify primary-study overlap across the rapidly expanding review corpus (11). The novelty of the present review is therefore its narrower exercise-based analytic boundary, inclusion of recent 2025 pairwise and network meta-analytic evidence, explicit separation of exercise-only, exercise-plus-nutrition and broader non-pharmacological evidence, AMSTAR-2 item-level appraisal and primary-study overlap quantification.

## Methods

### Design, registration and reporting

This umbrella review was prepared in accordance with PRISMA 2020 guidance and was prospectively registered in PROSPERO (CRD420261370155; https://www.crd.york.ac.uk/PROSPERO/view/CRD420261370155) (12). The review synthesized published systematic reviews with quantitative synthesis; therefore, ethics approval and informed consent were not required. The article type was defined as a Systematic Review for Frontiers in Nutrition, and the research question was framed around population, intervention, comparator, outcomes and study design (PICOS).

### Eligibility criteria and PICOS

The population of interest was middle-aged and/or older adults with SO as defined by the included review authors. For screening, middle-aged was operationalized pragmatically as adults aged 50 years or older, or as older-dominant samples in which the review reported some participants below 60 years; reviews restricted to adults aged 60 years or older were classified as older-adult reviews. The intervention of interest was exercise-based management, including exercise-only programs, exercise-modality comparisons and exercise-centered interventions combined with nutritional supplementation when the exercise-related quantitative synthesis was reported separately. Eligible comparators included usual care, no intervention, alternative exercise modalities, nutritional co-interventions or other comparators used by the included reviews. The primary outcome hierarchy comprised body composition and physical function. Body composition outcomes included adiposity indicators, body weight, BMI, appendicular skeletal muscle mass (ASM), appendicular skeletal muscle mass index (ASMI), fat-free mass and related indices. Physical-function outcomes included grip strength, gait speed, timed up-and-go, chair-rise or chair-stand performance, knee-extension strength and other mobility-related outcomes. Metabolic and inflammatory outcomes were treated as secondary and exploratory.

Eligible study designs were systematic reviews with pairwise meta-analysis and/or network meta-analysis of exercise-based intervention trials. Reviews with broader non-pharmacological scope were retained only when exercise-based quantitative syntheses were separately extractable for the primary umbrella analysis. Exercise-only and exercise-modality estimates were distinguished from exercise-plus-nutrition estimates where the source review reported them separately; nutrition-only, electroacupuncture or electrical-stimulation, and other non-exercise estimates were excluded. Reviews whose quantitative effect estimates combined randomized and non-randomized or quasi-experimental designs without separable randomized-trial estimates were not entered into the strict main pool. Accordingly, in Yin et al. (13) only the exercise-based and exercise-plus-nutrition syntheses were retained, while nutrition-only and electroacupuncture syntheses were excluded from the main analysis. Hita-Contreras et al. (14) was retained because its pooled syntheses were exercise-centered. A complete retained/excluded comparison table is provided in Supplementary Table S3, and the full-text exclusion and near-miss eligibility table is provided in Supplementary Table S4. We excluded umbrella reviews, narrative reviews, protocols, reviews without quantitative synthesis and reviews focused on broader or adjacent phenotypes such as sarcopenic overweight or osteosarcopenic obesity when SO-specific exercise-based quantitative data were not extractable.

### Information sources and search strategy

PubMed, Web of Science, SPORTDiscus, Cochrane Library, Embase and Scopus were searched from database inception to 1 March 2026. The search strategy combined terms for sarcopenic obesity, obesity-related sarcopenia, exercise-based and exercise-centered interventions, nutrition-related adjuncts, other non-pharmacological interventions and quantitative evidence-synthesis designs, including systematic reviews, pairwise meta-analyses and network meta-analyses. Age restrictions were not applied at database level to minimize the risk of missing retirement-age or older-dominant SO reviews; age and phenotype eligibility were enforced during screening and full-text assessment. Reference lists of eligible reviews and near-miss full-text reviews were also checked to improve search completeness. Full electronic search strategies and database-specific hit counts are provided in Supplementary Table S1.

### Selection process

**Figure 1:**
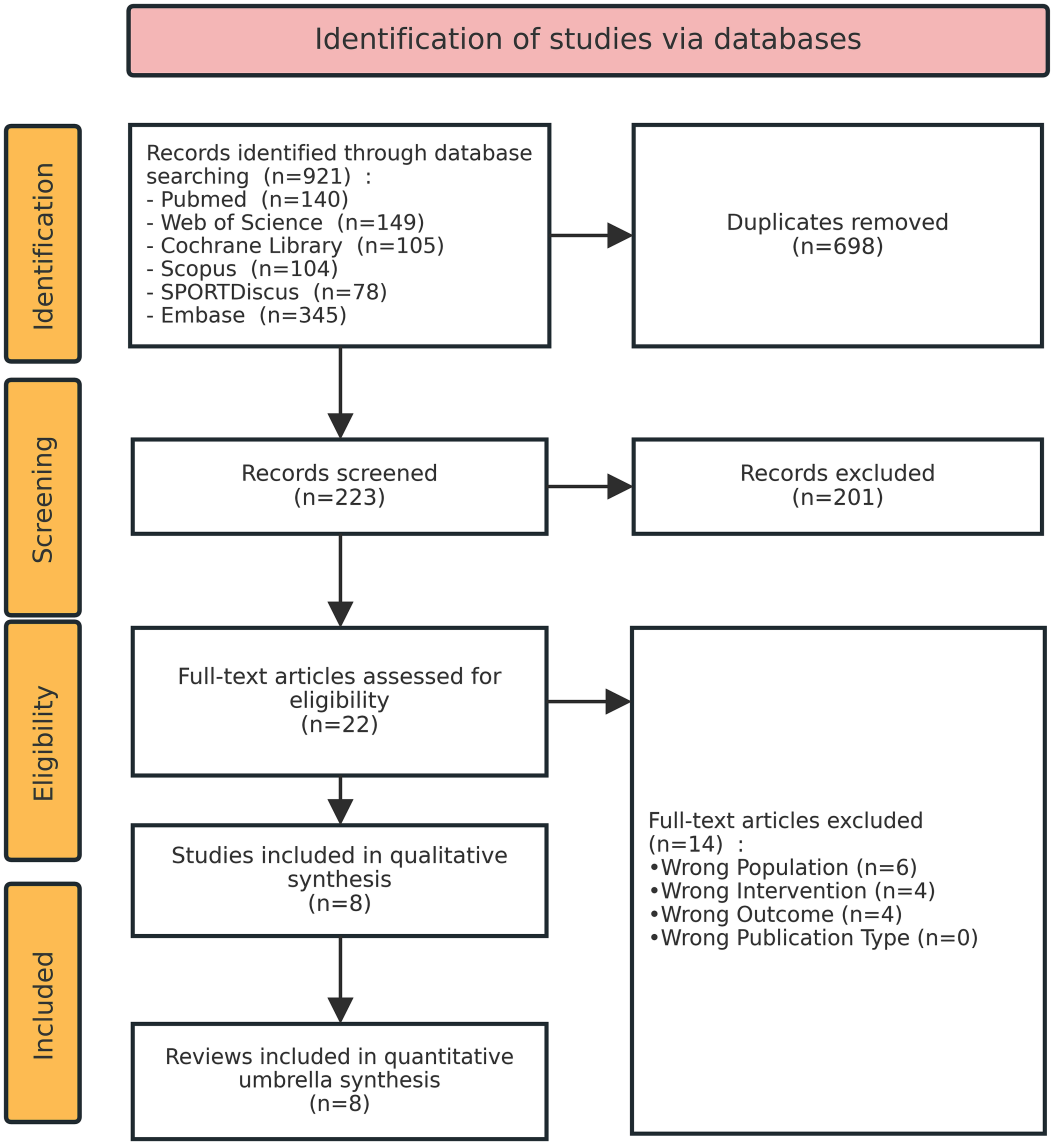
PRISMA flow diagram of study selection for the exercise-based strict main pool.

All records were imported into Zotero 8.0 and de-duplicated using the Duplicate Items function, followed by manual verification based on DOI, title, authors, journal and publication year to identify remaining duplicate or near-duplicate records. Two reviewers independently screened titles and abstracts and then independently assessed potentially eligible full texts against the predefined eligibility criteria. Disagreements at either stage were resolved by discussion. When consensus could not be reached, a third reviewer adjudicated. Screening decisions were made at review level, but eligibility for the strict main pool required that exercise-based quantitative estimates were either the review’s primary synthesis or separately extractable from broader non-pharmacological syntheses. Reviews with mixed intervention networks or pooled estimates that combined exercise modalities with nutrition-only or other non-exercise interventions were not entered into the strict exercise-based main pool unless exercise-based estimates could be isolated without changing the original synthesis structure. The study selection process is summarized in Figure 1.

### Data extraction and verification

Two reviewers independently extracted review-level data using a standardized extraction form. Extracted items included review design, population scope, SO definition or diagnostic criteria, age range or age threshold, intervention scope, comparator type, search end date, number of included primary studies, number of primary-study entries retained for the overlap matrix, participant characteristics as reported by each review, outcome domains, effect measures, units, direction considered favorable, pooled estimates, confidence or credible intervals, heterogeneity statistics and selected methodological features. All retained reviews were rechecked against their full texts to verify eligibility, estimate extraction and consistency of outcome labels. Related reports from the same underlying trial program were flagged during the overlap audit so that their influence could be interpreted conservatively and not used as a basis for statistical re-pooling.

### Methodological quality appraisal

Methodological quality was assessed independently by two reviewers using AMSTAR-2 (15). Item-level disagreements and overall-confidence disagreements were resolved by discussion, with third-reviewer adjudication when required. AMSTAR-2 was used to rate confidence in the conduct and reporting of included systematic reviews; it was not treated as a primary-trial risk-of-bias or outcome-level certainty tool. Item-level ratings for all 16 AMSTAR-2 items across the eight reviews are provided in Supplementary Table S5. Because the present umbrella review synthesized published review-level estimates and did not independently reappraise every primary trial, formal *de novo* GRADE ratings, JBI evidence ratings, or CINeMA ratings for the network component were not assigned. Applying those frameworks would have required reconstruction of the primary-trial evidence base, including trial-level risk of bias, inconsistency, indirectness, imprecision and publication-bias judgments across overlapping reviews. Instead, conservative umbrella-level certainty qualifiers were added for each outcome domain, explicitly anchored in review quality, consistency, primary-study overlap, heterogeneity and evidence sparsity.

### Primary-study overlap assessment

Primary-study overlap was quantified using a binary review-by-primary-study-entry matrix and the corrected covered area (CCA) (16). Author-year variants were harmonized, and duplicate reports or related publications from the same trial program were flagged during extraction to preserve traceability across source reviews. The CCA was calculated as (N - r)/(r × c - r), where *N* is the total number of primary-study occurrences across reviews, *r* is the number of unique primary-study entries and c is the number of reviews. Pairwise shared-entry counts, Jaccard indices and overlap coefficients were additionally calculated as descriptive overlap diagnostics. CCA values were interpreted descriptively using the thresholds proposed by Pieper et al. (16), with values greater than 15% indicating very high overlap.

### Outcome classification and review-level certainty

Evidence-direction labels were assigned using predefined descriptive rules rather than simple vote-counting. An outcome was classified as favorable when the extracted pooled or network estimate was statistically significant in the expected clinical direction and the confidence or credible interval excluded the null value. It was classified as generally favorable when most contributing estimates were favorable but at least one estimate was non-significant, differently scaled or less consistent. It was classified as mixed when statistically significant and non-significant or directionally discordant findings coexisted across reviews or outcome variants. It was classified as limited/inconsistent when few reviews contributed data, estimates were sparse, biomarkers were heterogeneous, or the available evidence was not based on between-group treatment effects. For each domain we considered effect size magnitude where interpretable, 95% confidence or credible intervals, I2 or other heterogeneity statistics, the direction considered favorable, AMSTAR-2 confidence and the degree of primary-study overlap. Minimal clinically important differences were considered conceptually but were not applied as formal thresholds because source reviews did not consistently report MCIDs and effect measures differed across outcomes.

Review-level certainty was rated conservatively as low or very low. A low rating was used when at least four reviews contributed broadly concordant review-level findings but certainty was limited by AMSTAR-2 confidence and very high overlap. A very-low rating was used when evidence was sparse, inconsistent, driven mainly by secondary biomarkers, based on within-group pre-post estimates, or dependent on network rankings with sparse direct comparisons. These labels are conservative umbrella-level certainty qualifiers, not formal *de novo* GRADE, JBI or CINeMA ratings, and should not be interpreted as primary-trial certainty.

### Why review-level estimates were not statistically re-pooled

The unit of synthesis was the published review-level estimate. We extracted and tabulated the effect measure used by each included review, including mean difference (MD), weighted mean difference (WMD), standardized mean difference (SMD) and network meta-analysis estimates where applicable. Outcome labels were harmonized only for presentation; effect measures were not converted across scales unless the original review had already done so. Heterogeneity statistics were reported when available from the source review.

Because primary-study overlap was very high, review-level pooled estimates were not statistically re-pooled. This decision avoided double counting of primary trials and preserved the distinction between an umbrella review and a new meta-analysis of primary studies. Instead, evidence direction was summarized descriptively as consistently favorable, generally favorable, mixed or limited/inconsistent. Descriptive subgroup and sensitivity summaries were interpreted as exploratory. Funnel-style or forest-style review-level displays, where used, were not treated as formal publication-bias tests or new meta-analytic syntheses because the number of reviews was small, effect measures differed and primary-study overlap was extensive.

Results are therefore expressed conservatively. We use terms such as reported favorable effects or directionally favorable when referring to published pooled estimates, and we avoid causal, optimal-modality or superiority claims unless these were directly supported by the included review design. Differences between resistance, aerobic, combined and multicomponent exercise should be read as hypothesis-supporting rather than definitive comparative-effectiveness conclusions.

## Results

### Search and selection

The PRISMA audit identified 921 records across the six-database corpus. After removal of 698 duplicate or near-duplicate records before screening, 223 titles and abstracts were screened, of which 201 were excluded. Twenty-two full-text articles were assessed for eligibility, and 14 were excluded for predefined reasons, most commonly because they were not systematic reviews with quantitative synthesis, did not focus on SO, did not report exercise-based extractable estimates, or addressed broader mixed non-pharmacological or exercise–nutrition networks outside the strict exercise-based main pool. Eight systematic reviews met the strict exercise-based eligibility criteria and were included in the final main pool: Hita-Contreras et al. (14), Yin et al. (13), Zhuang et al. (17), Chen et al. (18), Guo et al. (19), Polo-Ferrero et al. (20), Qiu et al. (21) and Wei et al. (22). The citation-level full-text exclusion and near-miss eligibility decisions are provided in Supplementary Table S4, and the selection process is shown in Figure 1.

### Characteristics of included reviews

The strict main pool comprised seven pairwise meta-analyses and one network meta-analysis published between 2018 and 2025 (13, 14, 17–22). Together, these reviews represented 33 unique primary-study units and 88 review-level primary-study occurrences. Review foci included exercise alone or exercise combined with nutritional supplementation, exercise-based subsets extracted from broader non-pharmacological reviews, exercise-modality comparisons, resistance-training-only syntheses, one exercise-modality network meta-analysis and one Stage I SO review. Stage I SO denotes SO without overt SO-related complications in the ESPEN/EASO staging framework, as applied by Wei et al. (22). The numbers of participants reported by individual reviews ranged from 303 to 955; these values were not summed because of extensive overlap in underlying primary studies. Review characteristics and SO diagnostic framing are summarized in Table 1, with extended details including search end date in Supplementary Table S2. Diagnostic comparability across reviews was limited. Chen et al. (18) and Wei et al. (22) were the only reviews that explicitly used an ESPEN/EASO-based framework, although Wei et al. (22) further restricted eligibility to Stage I, complication-free SO. The remaining reviews largely accepted author-defined or primary-trial SO criteria, with heterogeneous adiposity, muscle-mass and functional thresholds; therefore, cross-review differences in findings should not be attributed solely to exercise modality.

**Table 1:** Characteristics of the eight included exercise-based reviews.

Review	Design	Focus	Articles in review	Retained primary-study units	Participants reported	Population/age scope	SO definition/diagnostic framing as reported
Hita-Contreras et al. (14)	Pairwise MA	Exercise +/− nutrition (exercise-based pool)	9	9	558	Community-dwelling elderly adults aged ≥60 years	Author-defined SO in community-dwelling adults; criteria varied across included RCTs.
Yin et al. (13)	Pairwise MA	Exercise-based subset from broader non-pharmacological review	16	12	863	Adults with SO; included trial participants ranged 41–90 years (mean approximately 72 years)	Review accepted SO as defined by included trials; definitions were heterogeneous.
Zhuang et al.(17)	Pairwise MA	Exercise modality comparison	12	12	614	Older people with SO; pooled age range 58.4–88.4 years	Older people with SO; primary trials used heterogeneous body-composition and functional criteria.
Chen et al. (18)	Pairwise MA	Exercise modality comparison	8	8	424	SO patients in eight RCTs, predominantly older samples (approximately 68–81 years)	ESPEN/EASO 2022 SO criteria specified by the review.
Guo et al. (19)	Pairwise MA	RT only	7	7	303	Older females aged ≥60 years	Older females with SO; review-level diagnosis followed primary-trial SO criteria.
Polo-Ferrero et al. (20)	Pairwise MA	RT only	11	11	513	Adults >60 years with SO	Adults >60 years with SO; primary-trial diagnostic thresholds varied.
Qiu et al. (21)	NMA	Exercise modality NMA	14	14	955	Participants ≥60 years with SO	Participants ≥60 years with SO; NMA accepted SO definitions from included trials.
Wei et al. (22)	Pairwise MA	Stage I exercise	15	15	623	Adults aged ≥60 years with stage I, complication-free SO	Stage I SO under ESPEN/EASO staging: SO without overt SO-related complications.

MA, meta-analysis; NMA, network meta-analysis; RT, resistance training; SO, sarcopenic obesity. Yin et al. (13) was retained by partial extraction of exercise-based and exercise-plus-nutrition syntheses only. Participant counts are shown as reported by each review and were not summed because primary-study overlap was extensive. SO diagnostic framing was extracted at review level and may reflect heterogeneous primary-trial definitions.

### Methodological quality and overlap

Methodological quality was unfavorable overall. One review was rated critically low and seven were rated low confidence by AMSTAR-2 (Table 2; Supplementary Table S5). The most frequent critical weakness was failure to provide a citation-level list of excluded studies. Common non-critical weaknesses concerned limited justification for the choice of eligible study designs (I3), incomplete reporting of funding sources for included primary studies (I10), and limited evaluation of how primary-study risk of bias affected pooled results (I12). The repeated I7/I3/I10/I12 pattern therefore reflected a recurring reporting pattern in the SO review literature, especially the absence of citation-level excluded-study lists with justifications, rather than default scoring; the complete item-level matrix is provided in Supplementary Table S5.

**Table 2:** Methodological quality and overlap summary.

Review	AMSTAR-2 confidence	Critical flaw items	Non-critical weakness items
Hita-Contreras et al. (14)	Critically low	I2, I7	I3, I10, I12
Yin et al. (13)	Low	I7	I3, I10, I12
Zhuang et al.(17)	Low	I7	I3, I10, I12
Chen et al. (18)	Low	I7	I3, I10, I12
Guo et al. (19)	Low	I7	I3, I10, I12
Polo-Ferrero et al. (20)	Low	I7	I3, I10, I12
Qiu et al. (21)	Low	I7	I3, I10, I12
Wei et al. (22)	Low	I7	I3, I10, I12
Overall overlap	CCA = 0.238 (very high)	33 unique primary-study units; 88 primary-study occurrences	Highest raw overlap: Qiu et al. (21) vs. Wei et al. (22) (10 shared studies); highest Jaccard: Chen et al. (18) vs. Zhuang et al. (17) (0.667); Guo et al. (19) vs. Polo-Ferrero et al. (20) also high (0.636) owing to shared RT-only scope.

CCA, corrected covered area. Table 2 reports rating-driving No judgments for AMSTAR-2 critical-flaw and non-critical-weakness items; full Y/PY/N/NA judgments for all 16 AMSTAR-2 items, including partial-yes and not-applicable judgments, are provided in Supplementary Table S5. AMSTAR-2 item numbers follow the published AMSTAR-2 checklist. Overall overlap was calculated at primary-study level using the review-by-primary-study matrix.

Primary-study overlap was very high. The review-by-primary-study matrix included 33 unique primary-study units and 88 primary-study occurrences across eight reviews, yielding a CCA of 0.238. The most frequently repeated primary-study units were (42) and (43), each appearing in seven reviews. The highest raw pairwise overlap was observed for Qiu et al. (21) versus Wei et al. (22), with 10 shared primary studies, while the highest Jaccard index was observed for Chen et al. (18) versus Zhuang et al. (17) (0.667). Guo et al. (19) and Polo-Ferrero et al. (20) also showed high overlap (Jaccard = 0.636), reflecting their shared resistance-training-only scope. The overlap calculation summary, review-by-primary-study matrix and pairwise overlap diagnostics are provided in Supplementary Tables S6–S8.

### Body composition outcomes

**Figure 2:**
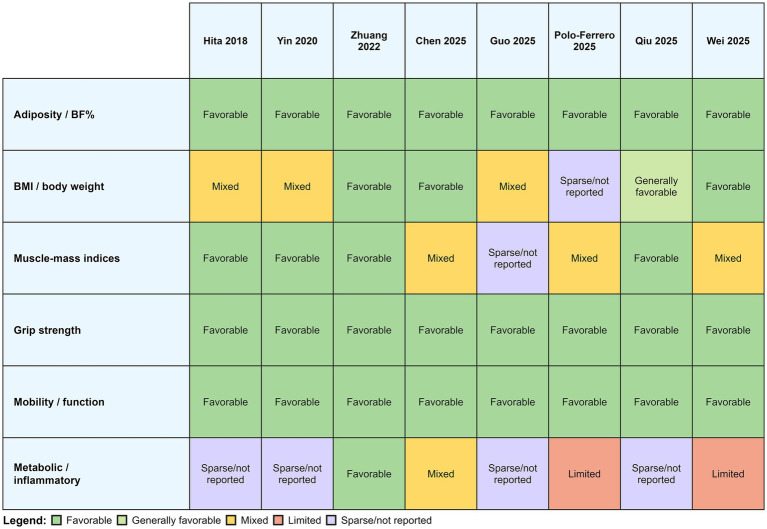
Direction and consistency of umbrella evidence across the main outcome hierarchy. The evidence map summarizes review-level interpretation for adiposity, BMI/body weight, muscle-mass indices, grip strength, mobility/functional performance, and metabolic/inflammatory outcomes using predefined umbrella-level classification rules. It is a descriptive umbrella map and does not represent statistical re-pooling.

Body composition evidence was most consistent for adiposity-related outcomes. All eight reviews reported favorable adiposity findings for body fat percentage or closely related fat measures (Table 3; Figure 2). Representative published estimates included MD −0.85 percentage points in Hita-Contreras et al. (14), MD −1.08 percentage points in the retained exercise-based synthesis of Yin et al. (13), MD −2.48 percentage points for any exercise and MD −2.96 percentage points for resistance training in Chen et al. (18), WMD −2.83 percentage points in Guo et al. (19) and MD −0.52 percentage points in Wei et al. (22). In the network meta-analysis by Qiu et al. (21), multicomponent training was placed highest for body-fat reduction by SUCRA ranking, but this ranking was interpreted as hypothesis-generating because it depends on network assumptions, sparse direct contrasts and overlap with other reviews (21).

**Table 3:** Summary of umbrella evidence for the main outcome hierarchy.

Outcome category	Reviews contributing	Direction of umbrella evidence	Representative published pooled estimates	Umbrella certainty qualifier	Interpretation
Adiposity/BF%	8	Consistently favorable	MD/WMD in percentage points: Hita-Contreras et al. (14) –0.85; Zhuang et al. (17) –1.08; Zhuang et al. (17) RT −2.96; Guo et al. (19) –2.83; Qiu et al. (21) MCT −6.37; Wei et al. (22) CE −0.68.	Low	Exercise-based interventions consistently reported reduced adiposity-related outcomes, but certainty was limited by low AMSTAR-2 confidence and very high overlap.
BMI / body weight	7	Generally favorable	BMI: Zhuang et al. (17) AT SMD −0.69 and mixed training SMD −0.77; Wei et al. (22) MD −1.35 kg/m^2^ overall. Body weight: Chen et al. (18) MD −3.45 kg overall and AT MD −6.07 kg.	Very low to low	BMI/body weight tended to improve most consistently under aerobic, combined or multicomponent approaches; resistance-only BMI effects were less uniform.
Muscle-mass indices	6	Mixed/inconsistent	Hita-Contreras et al. (14) ASM MD + 0.40 kg; Yin et al. (13) exercise + nutrition ASM MD + 0.43 kg; Zhuang et al. (17) ASMI SMD + 0.70 to +0.72; Qiu et al. (21) FFM MD + 5.21; Wei et al. (22) found no clear ASM/ASMI change.	Very low	Muscle-mass outcomes were directionally positive in some reviews but were not uniformly replicated.
Grip strength	6	Consistently favorable	MD in kg or SMD as reported: Hita-Contreras et al. (14) MD + 1.30 kg; Yin et al. (13) MD + 1.63 kg; Chen et al. (18) RT MD + 3.85 kg; Qiu et al. (21) RT SMD + 0.84; Wei et al. (22) RT MD + 3.43 kg.	Low	Resistance-focused programs showed the most consistent gains in grip strength.
Mobility/functional performance	8	Consistently favorable	Gait/walking speed: Hita-Contreras et al. (14) MD + 0.05 m/s; Yin et al. (13) MD + 0.13 m/s; Zhuang et al. (17) SMD + 0.71; Chen et al. (18) MD + 0.20 m/s; Qiu et al. (21) MCT MD + 0.35 m/s; Wei et al. (22)5 MD + 0.88 as reported. Additional functional outcomes included Guo et al. (19) TUG WMD -2.23, where lower time was favorable, and chair-rise WMD + 5.20, where higher performance was favorable.	Low	Mobility and functional outcomes improved across most exercise-centered reviews, although effect measures differed.
Metabolic / inflammatory markers	4	Limited/inconsistent	Zhuang 2022 favorable IGF-1; Chen et al. (18) favorable IGF-1 but null IL-6; Polo-Ferrero et al. (20) biomarker composite null; Wei et al. (22) within-group exploratory signals only.	Very low	Secondary biological outcomes remained sparse, heterogeneous and insufficiently consistent for firm inference.

Displayed estimates are published pooled or network estimates reported by the included reviews and are shown for umbrella synthesis only. Effect measures were retained as reported by the source reviews and were not converted across scales; therefore, MD/WMD, SMD and network estimates should not be compared as if they were on a common metric. Estimates were not statistically re-pooled across reviews because of very high primary-study overlap. Direction-favorable depends on the original outcome coding, particularly for outcomes where lower values are favorable, such as timed up-and-go, and outcomes where higher values are favorable, such as strength or chair-rise performance. Certainty qualifiers are conservative umbrella-level judgments, not full de novo GRADE/JBI/CINeMA ratings. AT, aerobic training; BF%, body fat percentage; CE, combined exercise; FFM, fat-free mass; MCT, multicomponent training; RT, resistance training; TCR, timed chair-rise; TUG, timed up-and-go.

BMI and body-weight outcomes were generally favorable but less uniform than adiposity outcomes. Reviews that separated aerobic, combined or multicomponent interventions often reported more favorable BMI or weight changes than resistance-training-only syntheses. For example, Zhuang et al. (17) reported favorable SMDs for BMI under aerobic and mixed training, Chen et al. (18) reported weight reduction for any exercise and for aerobic and mixed training subgroups, and Wei et al. (22) reported a favorable BMI change for any exercise and combined exercise. Resistance-training-only reviews showed smaller or less consistent BMI changes (19, 20).

Muscle-mass outcomes were mixed. Some reviews reported favorable changes in ASM, ASMI, standardized skeletal muscle mass index or fat-free mass, including Hita-Contreras et al. (14), Yin et al. (13), Zhuang et al. (17) and Qiu et al. (21). However, other reviews reported no clear improvement for some muscle-mass indices, and Wei et al. (22) did not find consistent ASM or ASMI changes in the Stage I SO evidence. We therefore interpreted muscle-mass evidence as directionally promising but not consistently replicated. Detailed extracted estimates for body-composition outcomes are provided in Supplementary Table S9.

### Physical-function outcomes

Physical-function evidence was generally favorable, particularly for grip strength and mobility-related outcomes. Grip strength improved in six reviews. Representative estimates included MD + 1.30 kg in Hita-Contreras et al. (14), MD + 1.63 kg in the exercise-based synthesis of Yin et al. (13), MD + 3.85 kg for resistance training in Chen et al. (18) and MD + 3.43 kg for resistance training in Wei et al. (22). In the network meta-analysis, resistance training had the highest SUCRA rank for handgrip strength, while multicomponent training also had a favorable estimate; these rankings were treated as hypothesis-generating rather than definitive modality-ordering evidence (21).

Gait speed and mobility-related outcomes were favorable across reviews contributing such outcomes. Representative estimates included MD + 0.05 m/s in Hita-Contreras et al. (14), MD + 0.13 m/s in Yin et al. (13), SMD + 0.71 for mixed or combined training in Zhuang et al. (17), MD + 0.20 m/s for walking speed in Chen et al. (18), and MD + 0.35 m/s for the multicomponent-training node in Qiu et al. (21). Guo et al. (19) also reported favorable timed up-and-go and chair-rise outcomes in older females undergoing resistance training (19). Because outcome definitions and effect measures differed, these estimates were not pooled across reviews. Detailed extracted estimates for physical-function outcomes are provided in Supplementary Table S10.

### Secondary metabolic and inflammatory outcomes

Metabolic and inflammatory outcomes were sparse, heterogeneous and often underpowered. Zhuang et al. (17) and Chen et al. (18) reported favorable IGF-1 findings, but Chen et al. (18) reported no clear IL-6 effect and Polo-Ferrero et al. (20) reported a null biomarker composite estimate. Wei et al. (22) reported selected within-group pre-post signals for metabolic and inflammatory markers, but these were not equivalent to between-group treatment effects (22). These outcomes were therefore interpreted as hypothesis-generating rather than practice-defining. Detailed extracted estimates are provided in Supplementary Table S11.

### Supplementary subgroup and sensitivity summaries

Descriptive subgroup summaries supported the main interpretation. Resistance training and combined or multicomponent exercise showed the highest proportion of favorable primary-outcome estimates. Resistance training was most consistently aligned with strength-related outcomes, whereas combined or multicomponent exercise appeared more favorable when adiposity reduction and mobility outcomes were considered together. Restricting the corpus from the exercise-based strict main pool to a narrower exercise-only or exercise-modality subset did not materially change the main message: adiposity, grip strength and mobility remained the most consistent favorable domains, while muscle-mass evidence remained mixed. These subgroup and sensitivity summaries were descriptive and were not interpreted as independent statistical tests.

## Discussion

### Principal findings

This umbrella review found a clinically coherent but methodologically cautious signal: exercise-based interventions for middle-aged and older adults with sarcopenic obesity (SO) were associated with favorable review-level findings for adiposity and physical function, whereas muscle-mass outcomes were less consistent and metabolic or inflammatory outcomes remained uncertain. The most reproducible signals concerned body-fat percentage or related adiposity measures, grip strength and gait- or mobility-related performance. These are clinically relevant endpoints because SO is defined by both excess adiposity and impaired muscle health, and because strength and mobility often determine independence more directly than body composition alone. However, all outcome-level interpretations should be considered low or very-low certainty at umbrella level because AMSTAR-2 confidence was low or critically low, primary-study overlap was very high and SO definitions were heterogeneous. This exercise-based interpretation is intentionally narrower than prior umbrella or broader non-pharmacological treatment reviews, which may combine exercise with nutrition, stimulation or other modalities (11, 23).

The findings should be interpreted within the wider geriatric nutrition and exercise literature. Sarcopenia has been described as a progressive skeletal-muscle disorder with major consequences for falls, disability and mortality (24). Earlier conceptual work on SO emphasized that excess adiposity and sarcopenia can reinforce one another through reduced mobility, ectopic fat deposition, low-grade inflammation and impaired metabolic health (25, 26). Disease-related muscle loss is also amplified by obesity, diabetes and other chronic conditions that are common in later life (27). These mechanisms make it plausible that exercise interventions can improve function and adiposity even when short-term changes in appendicular muscle mass are modest.

### Interpretation within nutrition and exercise science

From a nutrition perspective, SO should not be understood as a purely mechanical problem of insufficient exercise. Aging muscle is characterized by anabolic resistance, meaning that the muscle-protein synthetic response to feeding and exercise may be blunted unless the exercise stimulus and protein supply are sufficient (28). Expert recommendations for older adults therefore emphasize adequate high-quality protein intake and the combination of nutrition with exercise to preserve muscle function (29, 30). The present umbrella review did not evaluate nutrition-only strategies as its primary question, but this physiology explains why exercise-plus-nutrition syntheses may be clinically relevant when they are clearly separable from nutrition-only estimates.

The distinction between weight loss, fat loss and functional preservation is important. In obese older adults, intentional weight loss without an exercise stimulus can reduce fat mass but may also risk loss of lean tissue and function. A large randomized trial in obese older adults showed that combined aerobic and resistance exercise during weight management produced broader improvements in physical function than either mode alone, while also supporting favorable body-composition change (31). This evidence is not SO-specific, but it supports the interpretation that combined or multicomponent programs may be preferable when adiposity reduction and mobility targets are pursued together.

Protein supplementation is another important boundary condition. Meta-analyses of resistance-training studies have shown that protein supplementation can augment training-related gains in lean mass and strength, although the size of benefit depends on baseline protein intake, training dose, age and other participant characteristics (32, 33). In the SO review corpus, exercise-plus-nutrition findings were generally directionally favorable, but they were too heterogeneous to isolate the independent contribution of nutritional supplementation. The appropriate interpretation is therefore supportive rather than definitive: nutrition may enhance exercise adaptation, but the present review does not prove stand-alone nutritional efficacy.

### Why function improved more consistently than muscle mass

A notable pattern was that strength and mobility improved more consistently than muscle-mass indices. This is plausible because neural adaptation, improved motor-unit recruitment, better intermuscular coordination and task-specific practice can improve strength and performance before measurable hypertrophy occurs. Broad reviews of older adults have shown that progressive resistance training improves strength and physical function, even when lean-mass effects are smaller or more variable (34, 35). In SO, this distinction matters because functional endpoints may be more sensitive to clinically meaningful exercise adaptation than short-term changes in ASM or ASMI.

Multicomponent programs may provide additional functional benefits by combining resistance, aerobic, balance and task-specific elements. Evidence from frail older adults and expert exercise recommendations supports multicomponent training when the goal includes gait, balance, fall-risk reduction and activities of daily living rather than strength alone (36, 37). In parallel, longitudinal data in older adults suggest that adverse body composition is associated with gait-speed decline, reinforcing the need to target both adiposity and functional capacity (38). These considerations are consistent with our finding that combined or multicomponent interventions were most favorable when adiposity and mobility were considered together.

The mixed muscle-mass findings should not be interpreted as evidence that resistance training is ineffective. Hypertrophic responses in older adults require sufficient intensity, progression, duration, supervision and nutritional support, and the magnitude of lean-mass gain is often smaller than the change in strength or functional performance (39, 40). Diagnostic heterogeneity also complicates interpretation: some reviews used ASM, others ASMI, fat-free mass or standardized skeletal muscle mass indices, and the same absolute change may have different clinical meaning depending on baseline adiposity and body size.

### Intervention-specific implications

Resistance training showed the clearest strength-related signal. This is consistent with the physiology of muscle-force production and with clinical priorities in sarcopenia, where strength and performance often predict adverse outcomes more directly than muscle mass alone (7, 8, 24). For SO, resistance training should be regarded as a core exercise modality when the primary target is grip strength, lower-limb strength, chair-rise performance or daily functional capacity, but the certainty of this review-level inference remains low.

Combined or multicomponent exercise appeared more favorable when adiposity and mobility were prioritized together. Aerobic components can increase energy expenditure and cardiometabolic demand, whereas resistance and functional components support strength, gait and mobility. The network meta-analysis by Qiu et al. (21) supported this distinction only at a hypothesis-generating level by placing multicomponent training highest for body-fat and gait outcomes, and resistance training highest for handgrip and chair-stand performance. However, these rankings share many underlying primary studies with pairwise reviews and depend on network transitivity, consistency and node definitions, so they should not be interpreted as definitive optimal-modality evidence.

The review also clarifies the role of exercise-plus-nutrition evidence. Hita-Contreras et al. (14) and the retained exercise-based strata from Yin et al. (13) provided clinically relevant information because exercise remained central to the intervention contrast. However, broader non-pharmacological syntheses can mix exercise, nutrition, electrical stimulation and other modalities. Our strict retention rule was therefore necessary to avoid attributing effects to exercise when the underlying pooled estimate was driven by non-exercise components.

Yu et al. (41) was considered as adjacent evidence in this context. That NMA compared exercise modalities, nutritional supplementation, high-protein intake and combined nodes in a single network of 24 randomized trials. Because RT, multicomponent training and other exercise nodes were embedded in the same network geometry as nutrition-only and high-protein nodes, extracting selected exercise nodes would risk treating nutrition-dependent indirect evidence as independent exercise-only estimates. We therefore excluded Yu et al. (41) from the strict exercise-based main corpus and overlap matrix, but cited it as broader mixed exercise-nutrition evidence supporting the need to keep exercise-only, exercise-plus-nutrition and nutrition-only evidence analytically separate.

### Methodological implications

The main methodological message is that more meta-analyses do not necessarily mean more independent evidence. The CCA of 0.238 indicates very high overlap, with a relatively small number of primary-study units repeatedly contributing to multiple reviews. Without explicit overlap assessment, clinicians may mistake repeated synthesis for independent replication (16). This problem is especially important in SO because the primary-study base is small, SO definitions vary and exercise protocols differ in modality, dose, supervision, duration and co-intervention structure.

AMSTAR-2 findings further reduced confidence in fine-grained comparative claims. No included review achieved moderate or high confidence, and the most common critical limitation was the absence of a citation-level excluded-study list (15). Several reviews also gave limited attention to primary-study funding sources and to the influence of risk of bias on pooled findings. These weaknesses do not invalidate the broad direction of the adiposity and functional findings, but they limit certainty about effect size, modality ranking and generalizability across SO definitions.

Our decision not to statistically re-pool review-level estimates was important for transparency. Re-pooling meta-analytic estimates from overlapping reviews would have double-counted primary trials and produced misleading precision. The descriptive evidence map, subgroup summaries and sensitivity displays should therefore be read as structured evidence presentation rather than as new pooled treatment effects. This conservative approach is less visually dramatic than a new meta-analysis, but it is better aligned with the data structure.

### Clinical and research implications

For practice, the evidence cautiously supports an exercise-centered management model for middle-aged and older adults with SO, but this should not be read as a high-certainty clinical guideline. Resistance training should form the foundation when strength and functional independence are the main targets. Combined or multicomponent programs may be considered when fat reduction, gait improvement and broader mobility goals are equally important. Clinicians should avoid promising uniform increases in muscle mass over short intervention periods and should instead monitor adiposity, grip strength, gait speed, chair-rise performance, adherence, safety and nutritional adequacy.

Future primary trials should use consensus-informed SO definitions, report diagnostic thresholds clearly, describe exercise dose with enough detail for replication and prespecify a core set of body-composition and functional outcomes. Longer follow-up is needed to determine whether short-term improvements in adiposity and function translate into lower disability, falls, metabolic complications or health-care use. Trials should also report adverse events, adherence, dropout, supervision, progression rules and nutrition background so that clinicians can reproduce interventions rather than merely identify broad exercise categories.

Future secondary reviews should avoid redundant duplication unless they answer a distinct clinical question. When new reviews are justified, they should provide citation-level excluded-study lists, report primary-study funding sources, evaluate the impact of risk of bias on pooled findings and quantify primary-study overlap with prior reviews. For network meta-analyses, transitivity assumptions, intervention-node definitions and overlap with pairwise reviews should be stated explicitly.

## Strengths and limitations

Strengths of this umbrella review include a strict exercise-based retention rule, six-database search, independent screening and extraction, AMSTAR-2 appraisal, CCA calculation and conservative interpretation of review-level estimates. The approach clarified which conclusions were truly exercise-based and prevented nutrition-only or other non-exercise interventions from driving the primary narrative.

Several limitations should be acknowledged. First, the strict main pool was deliberately narrow, which improved interpretability but excluded adjacent reviews that examined broader non-pharmacological or mixed exercise-nutrition management, including Yu et al. (41). Second, our unit of analysis was the published review-level estimate; we did not reconstruct all primary-study datasets or conduct a new primary-study meta-analysis. Third, effect measures, SO definitions and intervention protocols varied across reviews, preventing valid statistical re-pooling. Fourth, AMSTAR-2 appraised review quality rather than outcome-level certainty, and a formal *de novo* GRADE assessment, JBI evidence grading, or CINeMA assessment for the network component was not performed because these approaches would require reconstruction and reappraisal of every primary trial across overlapping reviews, including trial-level risk-of-bias and evidence-domain judgments. Instead, we applied conservative umbrella-level low/very-low certainty qualifiers, which should not be interpreted as formal GRADE, JBI or CINeMA ratings. Fifth, safety, adherence and dropout reporting was inconsistent across source reviews and could not be synthesized robustly. Sixth, the high overlap means that agreement across reviews should be read as consistency of published synthesis rather than independent confirmation.

## Conclusion

Exercise-based interventions for middle-aged and older adults with SO were associated with favorable but low-certainty review-level findings for adiposity and physical function, especially body-fat reduction, grip strength and gait- or mobility-related performance. Resistance training showed the most consistent strength-related signal, whereas combined or multicomponent exercise appeared more favorable when adiposity and mobility targets were considered together. Muscle-mass outcomes remained mixed, and metabolic or inflammatory outcomes remained uncertain. The findings support cautious exercise-centered management of SO, but modality-specific and ranking claims should remain hypothesis-generating because the available review corpus is methodologically weak and highly overlapping.

## Data Availability

The raw data supporting the conclusions of this article will be made available by the authors, without undue reservation.

## Glossary

AMSTAR-2: A Measurement Tool to Assess Systematic Reviews 2
ASM: appendicular skeletal muscle mass
ASMI: appendicular skeletal muscle mass index
AT: aerobic training
BF%: body fat percentage
BMI: body mass index
CCA: corrected covered area
CE: combined exercise
CI: confidence interval
GRADE: Grading of Recommendations Assessment Development and Evaluation
MCID: minimal clinically important difference
MD: mean difference
MCT: multicomponent training
NMA: network meta-analysis
PICOS: population intervention comparator outcomes and study design
PRISMA: Preferred Reporting Items for Systematic Reviews and Meta-Analyses
RT: resistance training
SMD: standardized mean difference
SO: sarcopenic obesity
SUCRA: surface under the cumulative ranking curve
WMD: weighted mean difference
