# Supplementary material for: Exercise-based interventions for sarcopenic obesity in middle-aged and older adults: an umbrella review of systematic reviews with pairwise meta-analyses and network meta-analyses
Source: Front Nutr. 2026 Jun 10;13:1859967. doi: 10.3389/fnut.2026.1859967 (PMC13291741; doi:10.3389/fnut.2026.1859967)

**Supplementary Appendices**

For: Exercise-based interventions for sarcopenic obesity in middle-aged and older adults: an umbrella review of systematic reviews with pairwise meta-analyses and network meta-analyses

Search run date: database inception to 1 March 2026.

Exercise-based retention rule: broader non-pharmacological reviews were retained only when exercise-based quantitative syntheses were reported separately and were extractable for the primary umbrella analysis. Nutrition-only, electroacupuncture/electrical-stimulation and other non-exercise estimates were excluded from the primary umbrella analysis.

# **Table S1. Per-database search yields and full electronic search strategies.**

| **Database** | **Records identified** | **Search strategy** |
| --- | --- | --- |
| PubMed | 140 | ("sarcopenic obesity"[Title/Abstract] OR "sarcopenic obese*"[Title/Abstract] OR "obese sarcopenia"[Title/Abstract] OR (("sarcopenia"[Title/Abstract]) AND obes*[Title/Abstract])) AND (intervention*[Title/Abstract] OR treatment*[Title/Abstract] OR therap*[Title/Abstract] OR management[Title/Abstract] OR nonpharmacological[Title/Abstract] OR "non-pharmacological"[Title/Abstract] OR exercise*[Title/Abstract] OR training[Title/Abstract] OR "resistance training"[Title/Abstract] OR "resistance exercise"[Title/Abstract] OR "aerobic training"[Title/Abstract] OR "aerobic exercise"[Title/Abstract] OR "combined training"[Title/Abstract] OR "concurrent training"[Title/Abstract] OR "multicomponent training"[Title/Abstract] OR "circuit training"[Title/Abstract] OR "physical activity"[Title/Abstract] OR diet*[Title/Abstract] OR nutrition*[Title/Abstract] OR protein*[Title/Abstract] OR "protein supplementation"[Title/Abstract] OR "amino acid*"[Title/Abstract] OR "vitamin D"[Title/Abstract] OR "whole-body electromyostimulation"[Title/Abstract] OR "WB-EMS"[Title/Abstract] OR electromyostimulation[Title/Abstract] OR "neuromuscular electrical stimulation"[Title/Abstract] OR NMES[Title/Abstract] OR "electrical stimulation"[Title/Abstract]) AND ("Meta-Analysis"[Publication Type] OR "Systematic Review"[Publication Type] OR meta-analysis[Title/Abstract] OR "meta analysis"[Title/Abstract] OR "network meta-analysis"[Title/Abstract] OR "network meta analysis"[Title/Abstract] OR "systematic review"[Title/Abstract]) NOT (protocol[Title] OR editorial[Publication Type] OR letter[Publication Type] OR comment[Publication Type]) |
| Web of Science Core Collection | 149 | TS=(("sarcopenic obesity" OR "sarcopenic obese*" OR "obese sarcopenia" OR (sarcopeni* NEAR/3 obes*)) AND (intervention* OR treatment* OR therap* OR management OR nonpharmacological OR "non-pharmacological" OR exercise* OR training OR "resistance training" OR "resistance exercise" OR "aerobic training" OR "aerobic exercise" OR "combined training" OR "concurrent training" OR "multicomponent training" OR "circuit training" OR "physical activity" OR diet* OR nutrition* OR protein* OR "protein supplementation" OR "amino acid*" OR "vitamin D" OR "whole-body electromyostimulation" OR "WB-EMS" OR electromyostimulation OR "neuromuscular electrical stimulation" OR NMES OR "electrical stimulation") AND ("systematic review" OR "meta-analysis" OR "meta analysis" OR "network meta-analysis" OR "network meta analysis")) |
| SPORTDiscus | 78 | (TI ("sarcopenic obesity" OR "sarcopenic obese*" OR "obese sarcopenia" OR ("sarcopenia" AND obes*)) OR AB ("sarcopenic obesity" OR "sarcopenic obese*" OR "obese sarcopenia" OR ("sarcopenia" AND obes*))) AND (TI ("non-pharmacological" OR nonpharmacological OR exercise* OR training OR "resistance training" OR "resistance exercise" OR "aerobic training" OR "aerobic exercise" OR "combined training" OR "concurrent training" OR "multicomponent training" OR "circuit training" OR "physical activity" OR diet* OR nutrition* OR protein* OR "protein supplementation" OR "amino acid*" OR "vitamin D" OR "whole-body electromyostimulation" OR "WB-EMS" OR electromyostimulation OR "neuromuscular electrical stimulation" OR NMES OR "electrical stimulation") OR AB ("non-pharmacological" OR nonpharmacological OR exercise* OR training OR "resistance training" OR "resistance exercise" OR "aerobic training" OR "aerobic exercise" OR "combined training" OR "concurrent training" OR "multicomponent training" OR "circuit training" OR "physical activity" OR diet* OR nutrition* OR protein* OR "protein supplementation" OR "amino acid*" OR "vitamin D" OR "whole-body electromyostimulation" OR "WB-EMS" OR electromyostimulation OR "neuromuscular electrical stimulation" OR NMES OR "electrical stimulation")) AND (TI ("systematic review" OR "meta-analysis" OR "meta analysis" OR "network meta-analysis" OR "network meta analysis") OR AB ("systematic review" OR "meta-analysis" OR "meta analysis" OR "network meta-analysis" OR "network meta analysis")) |
| Cochrane Library | 105 | #1 ("sarcopenic obesity" OR "sarcopenic obese*" OR "obese sarcopenia" OR (sarcopenia AND obes*)):ti,ab,kw #2 ("non-pharmacological" OR nonpharmacological OR exercise* OR training OR "resistance training" OR "resistance exercise" OR "aerobic training" OR "aerobic exercise" OR "combined training" OR "concurrent training" OR "multicomponent training" OR "circuit training" OR "physical activity" OR diet* OR nutrition* OR protein* OR "protein supplementation" OR "amino acid*" OR "vitamin D" OR "whole-body electromyostimulation" OR "WB-EMS" OR electromyostimulation OR "neuromuscular electrical stimulation" OR NMES OR "electrical stimulation"):ti,ab,kw #3 ("systematic review" OR "meta-analysis" OR "meta analysis" OR "network meta-analysis" OR "network meta analysis"):ti,ab,kw #4 #1 AND #2 AND #3 |
| Embase | 345 | (('sarcopenia'/exp AND 'obesity'/exp) OR ('sarcopenic obesity' OR 'sarcopenic obese*' OR 'obese sarcopenia' OR (sarcopenia AND obes*)):ti,ab) AND (('exercise'/exp OR 'resistance training'/exp OR 'aerobic exercise'/exp OR 'physical activity'/exp OR 'diet'/exp OR 'nutrition'/exp OR 'protein supplementation'/exp OR 'amino acid'/exp OR 'vitamin d'/exp OR 'electromyostimulation'/exp OR 'electric stimulation'/exp) OR ('non-pharmacological' OR 'non pharmacological' OR nonpharmacological OR exercise* OR training OR 'resistance training' OR 'resistance exercise' OR 'aerobic training' OR 'aerobic exercise' OR 'combined training' OR 'concurrent training' OR 'multicomponent training' OR 'circuit training' OR 'physical activity' OR diet* OR nutrition* OR protein* OR 'protein supplementation' OR 'amino acid*' OR 'vitamin D' OR 'whole-body electromyostimulation' OR 'WB-EMS' OR electromyostimulation OR 'neuromuscular electrical stimulation' OR NMES OR 'electrical stimulation'):ti,ab) AND (('systematic review'/exp OR 'meta analysis'/exp) OR ('systematic review' OR 'meta-analysis' OR 'meta analysis' OR 'network meta-analysis' OR 'network meta analysis'):ti,ab) |
| Scopus | 104 | TITLE-ABS-KEY(("sarcopenic obesity" OR "sarcopenic obese*" OR "obese sarcopenia" OR (sarcopenia AND obes*)) AND ("non-pharmacological" OR nonpharmacological OR exercise* OR training OR "resistance training" OR "resistance exercise" OR "aerobic training" OR "aerobic exercise" OR "combined training" OR "concurrent training" OR "multicomponent training" OR "circuit training" OR "physical activity" OR diet* OR nutrition* OR protein* OR "protein supplementation" OR "amino acid*" OR "vitamin D" OR "whole-body electromyostimulation" OR "WB-EMS" OR electromyostimulation OR "neuromuscular electrical stimulation" OR NMES OR "electrical stimulation") AND ("systematic review" OR "meta-analysis" OR "meta analysis" OR "network meta-analysis" OR "network meta analysis")) |

Age restrictions were not applied at database level. Age eligibility was enforced during screening and full-text assessment. Records were imported into Zotero 8.0 and de-duplicated using the Duplicate Items function, followed by manual verification based on DOI, title, authors, journal and publication year.

# **Table S2. Extended characteristics of included reviews.**

| **Review** | **Design** | **Search end date** | **Databases searched by source review** | **Articles in source review** | **Retained primary-study units** | **Participants reported** | **Age scope** | **SO definition / diagnostic framing** | **Retention note** |
| --- | --- | --- | --- | --- | --- | --- | --- | --- | --- |
| Hita-Contreras 2018 | Pairwise MA | 2018-04-15 | CINAHL; Cochrane Plus; PubMed; SCOPUS; Web of Science | 9 | 9 | 558 | Community-dwelling elderly adults aged >=60 years | Author-defined SO in community-dwelling adults; criteria varied across included RCTs. | All retained syntheses were exercise-centred (exercise alone or exercise plus dietary supplementation). |
| Yin 2020 | Pairwise MA | 2019-09 | Cochrane Library; Scopus; EMBASE; PsycINFO; CINAHL; PubMed | 16 | 12 | 863 | Adults with SO; included trial participants ranged 41-90 years (mean ~72 years) | Review accepted SO as defined by included trials; definitions were heterogeneous. | Partial retention: nutrition-only and electrical-acupuncture/amino-acid syntheses removed from the exercise-based pool. |
| Zhuang 2022 | Pairwise MA | 2021-11 | PubMed; Embase; Cochrane Library; Web of Science; CNKI; Wanfang Data | 12 | 12 | 614 | Older people with SO; pooled age range 58.4-88.4 years | Older people with SO; primary trials used heterogeneous body-composition and functional criteria. | Retained as exercise-modality comparison. |
| Chen 2025 | Pairwise MA | 2023-10 | Cochrane Library; Web of Science; PubMed; Embase; CBM; CNKI; Wanfang; VIP | 8 | 8 | 424 | SO patients in eight RCTs, predominantly older samples (~68-81 years) | ESPEN/EASO 2022 SO criteria specified by the review. | Retained as exercise-modality comparison. |
| Guo 2025 | Pairwise MA | 2024-06 | PubMed; Web of Science; Embase; Cochrane Library | 7 | 7 | 303 | Older females aged >=60 years | Older females with SO; review-level diagnosis followed primary-trial SO criteria. | Retained as resistance-training-only synthesis. |
| Polo-Ferrero 2025 | Pairwise MA | 2024-07 | PubMed (Medline); SCOPUS; Cochrane Library; Embase; EBSCO; Web of Science | 11 | 11 | 513 | Adults >60 years with SO | Adults >60 years with SO; primary-trial diagnostic thresholds varied. | Retained as resistance-training-only synthesis. |
| Qiu 2025 | NMA | 2024-03 | PubMed; Embase; Cochrane Library; Web of Science; Scopus (plus updated Google Scholar/reference search) | 14 | 14 | 955 | Participants >=60 years with SO | Participants >=60 years with SO; NMA accepted SO definitions from included trials. | Retained because the network compared exercise modalities without nutrition-only nodes. |
| Wei 2025 | Pairwise MA | 2024-07 | PubMed; Embase; Web of Science; Cochrane Library; EBSCO | 15 | 15 | 623 | Adults aged >=60 years with stage I, complication-free SO | Stage I SO under ESPEN/EASO staging: SO without overt SO-related complications. | Retained as Stage I exercise review. |

# **Table S3. Retained and excluded comparison rules.**

| **Source / review type** | **Included in primary umbrella pool** | **Excluded from primary umbrella pool** | **Rationale** |
| --- | --- | --- | --- |
| Hita-Contreras 2018 | Exercise alone; exercise plus dietary supplementation | No nutrition-only pooled estimates in retained synthesis | Retained because pooled syntheses were exercise-centered and exercise remained part of the intervention contrast. |
| Yin 2020 | Exercise-based syntheses; exercise plus nutrition syntheses where separately reported | Nutrition-only syntheses; electroacupuncture/electrical-stimulation or other non-exercise syntheses | Partial extraction prevents nutrition-only or stimulation effects from being attributed to exercise. |
| Qiu 2025 | Exercise-modality NMA nodes only | None from the source review for primary inclusion | Retained because the network compared exercise modalities without nutrition-only nodes. Rankings were interpreted cautiously. |
| Yu 2026 | None in the strict main pool | The full mixed exercise-nutrition NMA, including nutrition-only/high-protein/nutritional-supplementation nodes | Excluded from the primary corpus because exercise estimates were embedded in a mixed NMA network and could not be isolated as exercise-only review-level estimates without reconstructing the original primary-trial network. Cited as adjacent evidence in the Discussion. |
| Broader non-pharmacological reviews | Only exercise-based quantitative syntheses that were separately extractable | Narrative-only results, nutrition-only estimates, stimulation-only estimates, or broader intervention estimates without separable exercise strata | Rule applied to preserve the primary exercise-based analytic boundary. |

# **Table S4. Citation-level full-text exclusion and near-miss eligibility decisions.**

| **Full-text ID** | **Citation** | **Year** | **Title** | **Decision for strict main pool** | **Primary exclusion category** | **Specific reason** |
| --- | --- | --- | --- | --- | --- | --- |
| FTX01 | Zeng et al. 2025 | 2025 | Effects of non-pharmacological interventions on body composition and physical function in older women with sarcopenic obesity: a meta-analysis | Excluded from strict exercise-based main pool | Intervention boundary | Broader non-pharmacological intervention review; not retained because the strict main pool required exercise-based quantitative syntheses that could be isolated from non-exercise components. |
| FTX02 | Chen et al. 2025 (J Nursing Research) | 2025 | Effects of Resistance Exercise in Older Adults With Sarcopenic Obesity: A Systematic Review and Meta-Analysis | Excluded from strict exercise-based main pool | Outcome/review-scope boundary | Resistance-exercise review with mixed RCT/quasi-experimental source-design scope; not entered into the strict randomized-trial exercise-review pool because a separable RCT-only review-level estimate was not available from the source synthesis. |
| FTX03 | da Silva Gonçalves et al. 2023 | 2023 | The effects of different exercise training types on body composition and physical performance in older adults with sarcopenic obesity: a systematic review and meta-analysis | Excluded from strict exercise-based main pool | Population/source-design boundary | Exercise-focused review that included randomized and controlled clinical trials; excluded from the strict main pool because source-design eligibility was broader than the randomized-trial-focused corpus used for primary overlap reconciliation. |
| FTX04 | Eglseer et al. 2023 | 2023 | Nutritional and exercise interventions in individuals with sarcopenic obesity around retirement age: a systematic review and meta-analysis | Excluded from strict exercise-based main pool | Population/scope boundary | Mixed nutrition-exercise review in persons around retirement age (50-70 years); not retained in the strict older-dominant exercise-based main pool, but considered as adjacent evidence. |
| FTX05 | Hernandez-Martinez et al. 2025 | 2025 | Effects of concurrent training on biomarkers, morphological variables, and physical performance in people with sarcopenic obesity: a meta-analysis with meta-regression | Excluded from strict exercise-based main pool | Population boundary | Exercise-based review but eligible population was adults aged >=18 years and not specifically middle-aged/older adults with SO. |
| FTX06 | Hsu et al. 2019 | 2019 | Effects of exercise and nutritional intervention on body composition, metabolic health, and physical performance in adults with sarcopenic obesity: a meta-analysis | Excluded from strict exercise-based main pool | Population/scope boundary | Mixed exercise/nutrition review with broader intervention scope; not retained as a strict exercise-based review because nutrition-only and combined-intervention estimates could not be entered into the main exercise-only overlap corpus without altering the analytic boundary. |
| FTX07 | Reiter et al. 2023 | 2023 | Effects of nutrition and exercise interventions on persons with sarcopenic obesity: an umbrella review of meta-analyses of randomised controlled trials | Excluded from strict main pool; cited as prior umbrella review | Outcome/review-scope boundary | Umbrella review of meta-analyses rather than a source systematic review/meta-analysis; cited to differentiate novelty but excluded from the primary umbrella corpus. |
| FTX08 | Şengül Ayçiçek et al. 2025 | 2025 | Prevalence, diagnosis and treatment of sarcopenic obesity in older adults: a systematic review and a meta-analysis | Excluded from strict exercise-based main pool | Outcome/review-scope boundary | Review focused on prevalence, diagnosis and broad treatment rather than a strict exercise-based quantitative intervention synthesis. |
| FTX09 | Tian et al. 2024 | 2024 | Non-pharmacological treatment strategies for anthropometric, physical capacity and physiological indicators among sarcopenic obesity patients: a systematic review of rigorous randomized controlled trials | Excluded from strict exercise-based main pool | Population/scope boundary | Broader non-pharmacological review including adjacent phenotypes such as sarcopenic overweight as well as sarcopenic obesity; excluded from the strict SO exercise-based main pool. |
| FTX10 | Wang et al. 2026 | 2026 | Exercise, nutrition, physical agent therapy in older adults with sarcopenic obesity: a systematic review and network meta-analysis | Excluded from strict exercise-based main pool | Intervention boundary | Mixed rehabilitation NMA combining exercise with nutrition-only, physical-agent therapy, WBEMS and electroacupuncture nodes; exercise-only estimates could not be isolated without reconstructing the network. |
| FTX11 | Xu et al. 2023 (PLOS ONE) | 2023 | Effects of non-pharmacological interventions on patients with sarcopenic obesity: a meta-analysis | Excluded from strict exercise-based main pool | Outcome/review-scope boundary | Broader non-pharmacological synthesis rather than a strict exercise-based review corpus; separable exercise-based SO estimates were not used as a standalone main-pool evidence source. |
| FTX12 | Xu et al. 2025 (Nutrients) | 2025 | Exercise, nutrition, and neuromuscular electrical stimulation for sarcopenic obesity: a systematic review and meta-analysis of management in middle-aged and older adults | Excluded from strict exercise-based main pool | Population/scope boundary | Included adjacent populations such as sarcopenic overweight or obesity with sarcopenia risk and combined exercise with nutrition/NMES; outside strict SO exercise-based main-pool boundaries. |
| FTX13 | Yang et al. 2022 | 2022 | Effects of WB-EMS and protein supplementation on body composition, physical function, metabolism and inflammatory biomarkers in middle-aged and elderly patients with sarcopenic obesity: a meta-analysis of randomized controlled trials | Excluded from strict exercise-based main pool | Intervention boundary | Focused on WB-EMS and protein/amino-acid supplementation rather than exercise-based management; electrical stimulation and nutrition-only estimates were outside the primary analytic boundary. |
| FTX14 | Yu et al. 2026 | 2026 | Comparative effectiveness of exercise modalities and nutritional supplementation for sarcopenic obesity in older adults: a network meta-analysis based on randomized controlled trials | Excluded from strict main pool; cited as adjacent evidence | Intervention boundary | Mixed exercise-nutrition NMA; exercise nodes were embedded with nutrition-only/high-protein/nutritional-supplementation nodes and could not be isolated as exercise-only estimates without reconstructing the NMA. |

Reasons are reported for full-text records assessed but not entered into the strict exercise-based main pool. Yu et al. 2026 and Reiter et al. 2023 were cited as adjacent or prior evidence where relevant but not included in the primary CCA matrix.

# **Table S5. AMSTAR-2 item-level ratings.**

| **Review** | **Year** | **Journal** | **Overall confidence** | **I1** | **I2** | **I3** | **I4** | **I5** | **I6** | **I7** | **I8** | **I9** | **I10** | **I11** | **I12** | **I13** | **I14** | **I15** | **I16** |
| --- | --- | --- | --- | --- | --- | --- | --- | --- | --- | --- | --- | --- | --- | --- | --- | --- | --- | --- | --- |
| Hita-Contreras 2018 | 2018 | Maturitas | Critically low | Y | N | N | PY | Y | Y | N | Y | Y | N | Y | N | Y | Y | Y | Y |
| Yin 2020 | 2020 | Experimental Gerontology | Low | Y | Y | N | Y | Y | Y | N | Y | Y | N | Y | N | Y | Y | NA | Y |
| Zhuang 2022 | 2022 | Frontiers in Physiology | Low | Y | Y | N | Y | Y | Y | N | Y | Y | N | Y | N | Y | Y | NA | Y |
| Chen 2025 | 2025 | BMC Geriatrics | Low | Y | Y | N | Y | Y | Y | N | Y | Y | N | Y | N | Y | Y | Y | Y |
| Guo 2025 | 2025 | Frontiers in Aging Neuroscience | Low | Y | Y | N | PY | Y | Y | N | Y | Y | N | Y | N | Y | Y | NA | Y |
| Polo-Ferrero 2025 | 2025 | Nursing Reports | Low | Y | Y | N | Y | Y | Y | N | Y | Y | N | Y | N | Y | Y | Y | Y |
| Qiu 2025 | 2025 | Frontiers in Nutrition | Low | Y | Y | N | Y | Y | Y | N | Y | Y | N | Y | N | Y | Y | Y | Y |
| Wei 2025 | 2025 | Frontiers in Nutrition | Low | Y | Y | N | Y | Y | Y | N | Y | Y | N | Y | N | Y | Y | Y | Y |

Y, yes; PY, partial yes; N, no; NA, not applicable or not assessable from available source details.

# **Table S6. Primary-study overlap methods and summary.**

| **Overlap item** | **Value** |
| --- | --- |
| Number of included umbrella reviews | 8 |
| Number of unique primary-study units in overlap matrix | 33 |
| Total review-level primary-study occurrences | 88 |
| Corrected covered area (CCA) | 0.238 |
| CCA interpretation | Very high overlap (>15%) |
| Most frequently repeated primary-study units | Huang et al. (2017) and Liao et al. (2018), each appearing in seven reviews |
| Highest pairwise shared-study count | Qiu 2025 vs Wei 2025: 10 shared primary studies |
| Highest pairwise Jaccard index | Chen 2025 vs Zhuang 2022: 0.667 |
| High-scope-overlap RT-only pair | Guo 2025 vs Polo-Ferrero 2025: Jaccard 0.636, reflecting shared RT-only scope |

# Table S7. Binary review-by-primary-study overlap matrix.

| **primary_study** | **Chen et al. (2025)** | **Guo et al. (2025)** | **Hita-Contreras et al. (2018)** | **Polo-Ferrero et al. (2025)** | **Qiu et al. (2025)** | **Wei et al. (2025)** | **Yin et al. (2020)** | **Zhuang et al. (2022)** |
| --- | --- | --- | --- | --- | --- | --- | --- | --- |
| Balachandran et al. (2014) | 0 | 0 | 0 | 0 | 0 | 1 | 1 | 0 |
| Gadelha et al. (2016) | 0 | 0 | 1 | 0 | 1 | 0 | 1 | 0 |
| Kemmler et al. (2016) | 0 | 0 | 1 | 0 | 0 | 0 | 1 | 0 |
| Kim et al. (2016) | 1 | 0 | 1 | 0 | 1 | 1 | 1 | 1 |
| Maltais et al. (2016) | 0 | 0 | 0 | 0 | 0 | 1 | 0 | 0 |
| Muscariello et al. (2016) | 0 | 0 | 0 | 0 | 0 | 0 | 1 | 0 |
| Vasconcelos et al. (2016) | 1 | 1 | 0 | 1 | 1 | 1 | 0 | 1 |
| Chen et al. (2017) | 1 | 0 | 1 | 0 | 1 | 1 | 1 | 1 |
| Huang et al. (2017) | 1 | 1 | 1 | 1 | 1 | 1 | 0 | 1 |
| Kemmler et al. (2017) | 0 | 0 | 1 | 0 | 0 | 0 | 1 | 0 |
| Liao et al. (2017) | 1 | 1 | 0 | 1 | 1 | 1 | 0 | 1 |
| Park et al. (2017) | 1 | 0 | 1 | 0 | 1 | 1 | 1 | 1 |
| Sammarco et al. (2017) | 0 | 0 | 0 | 0 | 0 | 0 | 1 | 0 |
| Chiu et al. (2018) | 0 | 0 | 0 | 0 | 1 | 1 | 0 | 1 |
| Cunha et al. (2018) | 0 | 1 | 0 | 1 | 0 | 0 | 0 | 0 |
| Kemmler et al. (2018a) | 0 | 0 | 1 | 0 | 0 | 0 | 0 | 0 |
| Kemmler et al. (2018b) | 0 | 0 | 1 | 0 | 0 | 0 | 0 | 0 |
| Liao et al. (2018) | 1 | 1 | 0 | 1 | 1 | 1 | 1 | 1 |
| Zhou et al. (2018) | 0 | 0 | 0 | 0 | 0 | 0 | 1 | 0 |
| Nabuco et al. (2019) | 0 | 0 | 0 | 0 | 0 | 1 | 1 | 0 |
| Wang et al. (2019) | 1 | 0 | 0 | 0 | 1 | 0 | 0 | 1 |
| Banitalebi et al. (2020) | 0 | 1 | 0 | 1 | 0 | 0 | 0 | 0 |
| Hashemi et al. (2020) | 0 | 0 | 0 | 1 | 0 | 0 | 0 | 0 |
| Li et al. (2020) | 0 | 0 | 0 | 0 | 0 | 0 | 0 | 1 |
| Liao et al. (2020) | 0 | 0 | 0 | 0 | 0 | 1 | 0 | 0 |
| Marcos-Pardo et al. (2020) | 0 | 0 | 0 | 0 | 1 | 0 | 0 | 0 |
| Banitalebi et al. (2021) | 0 | 0 | 0 | 1 | 0 | 0 | 0 | 1 |
| Lee et al. (2021) | 0 | 1 | 0 | 1 | 0 | 0 | 0 | 1 |
| Hashemi et al. (2022) | 0 | 0 | 0 | 1 | 0 | 0 | 0 | 0 |
| Jung et al. (2022) | 0 | 0 | 0 | 1 | 1 | 1 | 0 | 0 |
| Ferhi et al. (2023) | 0 | 0 | 0 | 0 | 1 | 0 | 0 | 0 |
| Magtouf et al. (2023) | 0 | 0 | 0 | 0 | 1 | 1 | 0 | 0 |
| Jung et al. (2024) | 0 | 0 | 0 | 0 | 0 | 1 | 0 | 0 |

# Table S8. Pairwise overlap diagnostics calculated from the overlap matrix.

| **Review A** | **Review B** | **Shared primary studies** | **Jaccard index** | **Overlap coefficient** | **Shared primary-study labels** |
| --- | --- | --- | --- | --- | --- |
| Qiu (2025) | Wei (2025) | 10 | 0.526 | 0.714 | Chen (2017) \| Chiu (2018) \| Huang (2017) \| Jung (2022) \| Kim (2016) \| Liao (2017) \| Liao (2018) \| Magtouf (2023) \| Park (2017) \| Vasconcelos (2016) |
| Qiu (2025) | Zhuang (2022) | 9 | 0.529 | 0.750 | Chen (2017) \| Chiu (2018) \| Huang (2017) \| Kim (2016) \| Liao (2017) \| Liao (2018) \| Park (2017) \| Vasconcelos (2016) \| Wang (2019) |
| Chen (2025) | Zhuang (2022) | 8 | 0.667 | 1.000 | Chen (2017) \| Huang (2017) \| Kim (2016) \| Liao (2017) \| Liao (2018) \| Park (2017) \| Vasconcelos (2016) \| Wang (2019) |
| Chen (2025) | Qiu (2025) | 8 | 0.571 | 1.000 | Chen (2017) \| Huang (2017) \| Kim (2016) \| Liao (2017) \| Liao (2018) \| Park (2017) \| Vasconcelos (2016) \| Wang (2019) |
| Wei (2025) | Zhuang (2022) | 8 | 0.421 | 0.667 | Chen (2017) \| Chiu (2018) \| Huang (2017) \| Kim (2016) \| Liao (2017) \| Liao (2018) \| Park (2017) \| Vasconcelos (2016) |
| Guo (2025) | Polo-Ferrero (2025) | 7 | 0.636 | 1.000 | Banitalebi (2020) \| Cunha (2018) \| Huang (2017) \| Lee (2021) \| Liao (2017) \| Liao (2018) \| Vasconcelos (2016) |
| Chen (2025) | Wei (2025) | 7 | 0.438 | 0.875 | Chen (2017) \| Huang (2017) \| Kim (2016) \| Liao (2017) \| Liao (2018) \| Park (2017) \| Vasconcelos (2016) |
| Hita-Contreras (2018) | Yin (2020) | 6 | 0.400 | 0.667 | Chen (2017) \| Gadelha (2016) \| Kemmler (2016) \| Kemmler (2017) \| Kim (2016) \| Park (2017) |
| Polo-Ferrero (2025) | Zhuang (2022) | 6 | 0.353 | 0.545 | Banitalebi (2021) \| Huang (2017) \| Lee (2021) \| Liao (2017) \| Liao (2018) \| Vasconcelos (2016) |
| Wei (2025) | Yin (2020) | 6 | 0.286 | 0.500 | Balachandran (2014) \| Chen (2017) \| Kim (2016) \| Liao (2018) \| Nabuco (2019) \| Park (2017) |
| Guo (2025) | Zhuang (2022) | 5 | 0.357 | 0.714 | Huang (2017) \| Lee (2021) \| Liao (2017) \| Liao (2018) \| Vasconcelos (2016) |
| Hita-Contreras (2018) | Qiu (2025) | 5 | 0.278 | 0.556 | Chen (2017) \| Gadelha (2016) \| Huang (2017) \| Kim (2016) \| Park (2017) |
| Polo-Ferrero (2025) | Qiu (2025) | 5 | 0.250 | 0.455 | Huang (2017) \| Jung (2022) \| Liao (2017) \| Liao (2018) \| Vasconcelos (2016) |
| Polo-Ferrero (2025) | Wei (2025) | 5 | 0.238 | 0.455 | Huang (2017) \| Jung (2022) \| Liao (2017) \| Liao (2018) \| Vasconcelos (2016) |
| Qiu (2025) | Yin (2020) | 5 | 0.238 | 0.417 | Chen (2017) \| Gadelha (2016) \| Kim (2016) \| Liao (2018) \| Park (2017) |
| Chen (2025) | Guo (2025) | 4 | 0.364 | 0.571 | Huang (2017) \| Liao (2017) \| Liao (2018) \| Vasconcelos (2016) |
| Chen (2025) | Hita-Contreras (2018) | 4 | 0.308 | 0.500 | Chen (2017) \| Huang (2017) \| Kim (2016) \| Park (2017) |
| Chen (2025) | Polo-Ferrero (2025) | 4 | 0.267 | 0.500 | Huang (2017) \| Liao (2017) \| Liao (2018) \| Vasconcelos (2016) |
| Chen (2025) | Yin (2020) | 4 | 0.250 | 0.500 | Chen (2017) \| Kim (2016) \| Liao (2018) \| Park (2017) |
| Guo (2025) | Qiu (2025) | 4 | 0.235 | 0.571 | Huang (2017) \| Liao (2017) \| Liao (2018) \| Vasconcelos (2016) |
| Hita-Contreras (2018) | Zhuang (2022) | 4 | 0.235 | 0.444 | Chen (2017) \| Huang (2017) \| Kim (2016) \| Park (2017) |
| Guo (2025) | Wei (2025) | 4 | 0.222 | 0.571 | Huang (2017) \| Liao (2017) \| Liao (2018) \| Vasconcelos (2016) |
| Hita-Contreras (2018) | Wei (2025) | 4 | 0.200 | 0.444 | Chen (2017) \| Huang (2017) \| Kim (2016) \| Park (2017) |
| Yin (2020) | Zhuang (2022) | 4 | 0.200 | 0.333 | Chen (2017) \| Kim (2016) \| Liao (2018) \| Park (2017) |
| Guo (2025) | Hita-Contreras (2018) | 1 | 0.067 | 0.143 | Huang (2017) |
| Guo (2025) | Yin (2020) | 1 | 0.056 | 0.143 | Liao (2018) |
| Hita-Contreras (2018) | Polo-Ferrero (2025) | 1 | 0.053 | 0.111 | Huang (2017) |
| Polo-Ferrero (2025) | Yin (2020) | 1 | 0.045 | 0.091 | Liao (2018) |

# **Table S9. Review-level pooled effects for primary body-composition outcomes.**

| **Review** | **Outcome** | **Intervention** | **Comparator** | **Metric** | **Effect** | **CI low** | **CI high** | **I2** | **Notes** | **Exercise-based pool note** |
| --- | --- | --- | --- | --- | --- | --- | --- | --- | --- | --- |
| Hita-Contreras 2018 | Appendicular skeletal muscle mass | EXE alone + EXE+SUPPL | Control | MD | 0.40 | 0.18 | 0.63 | 0% |  | Exercise-alone and exercise+supplement syntheses retained as exercise-based evidence. |
| Chen 2025 | Percentage body fat | Any exercise | Control | MD | -2.48 | -3.25 | -1.70 | 0% |  |  |
| Chen 2025 | Percentage body fat | Resistance training | Control | MD | -2.96 | -4.19 | -1.74 |  |  |  |
| Chen 2025 | Percentage body fat | Mixed training | Control | MD | -2.42 | -3.58 | -1.26 |  |  |  |
| Guo 2025 | Body fat percentage | Resistance training | Control | WMD | -2.83 | -4.55 | -1.12 | 51% |  |  |
| Hita-Contreras 2018 | Body fat percentage | EXE alone + EXE+SUPPL | Control | MD | -0.85 | -1.61 | -0.08 | 62% | Overall pooled across exercise-only and exercise+supplement arms; no significant subgroup differences. | Exercise-alone and exercise+supplement syntheses retained as exercise-based evidence. |
| Polo-Ferrero 2025 | Body fat percentage | Resistance training | No intervention | SMD | 0.52 | 0.19 | 0.86 |  | Direction interpreted as favorable by source review. |  |
| Qiu 2025 | Body fat percentage | Multicomponent training (MCT) | Control | MD | -6.37 | -8.67 | -4.07 |  | NMA estimate; MCT ranked highest for BFP. |  |
| Qiu 2025 | Body fat percentage | Combined resistance + aerobic training (CT) | Control | MD | -2.08 | -4.00 | -0.16 |  |  |  |
| Qiu 2025 | Body fat percentage | Resistance training (RT) | Control | MD | -1.85 | -3.25 | -0.44 |  |  |  |
| Wei 2025 | Body fat percentage | Any exercise | Control | MD | -0.52 | -0.72 | -0.33 |  |  |  |
| Wei 2025 | Body fat percentage | Combined exercise (CE) | Control | MD | -0.68 | -0.95 | -0.41 |  |  |  |
| Wei 2025 | Body fat percentage | Resistance training (RT) | Control | MD | -0.36 | -0.66 | -0.06 |  |  |  |
| Yin 2020 | Body fat percentage | Exercise-based intervention | Usual care / blank control | MD | -1.08 | -1.99 | -0.17 | 50% |  | Exercise-based and exercise+nutrition syntheses retained; nutrition-only and electroacupuncture syntheses excluded from the exercise-based pool. |
| Zhuang 2022 | Body fat percentage | Resistance training | No physical activity control | SMD | -0.43 | -0.63 | -0.22 | 38% |  |  |
| Zhuang 2022 | Body fat percentage | Mixed / combined training | No physical activity control | SMD | -0.54 | -0.83 | -0.25 | 0% |  |  |
| Guo 2025 | BMI | Resistance training | Control | WMD | -0.42 | -1.92 | 1.08 | 33% |  |  |
| Qiu 2025 | BMI | Multicomponent training (MCT) | Control | MD | 0.74 | 0.08 | 1.40 |  | Paper reports positive MD and interprets MCT as superior for BMI; likely due to network coding convention. |  |
| Wei 2025 | BMI | Any exercise | Control | MD | -1.35 | -1.99 | -0.70 |  |  |  |
| Wei 2025 | BMI | Combined exercise (CE) | Control | MD | -1.25 | -1.96 | -0.55 |  |  |  |
| Yin 2020 | BMI | Exercise-based intervention | Usual care / blank control | MD | -0.17 | -0.67 | 0.33 | 0% |  | Exercise-based and exercise+nutrition syntheses retained; nutrition-only and electroacupuncture syntheses excluded from the exercise-based pool. |
| Zhuang 2022 | BMI | Aerobic training | No physical activity control | SMD | -0.69 | -1.18 | -0.21 | 0% |  |  |
| Zhuang 2022 | BMI | Mixed / combined training | No physical activity control | SMD | -0.77 | -1.26 | -0.28 | 0% |  |  |
| Yin 2020 | Appendicular skeletal muscle mass | Exercise plus nutrition | Usual care / blank control | MD | 0.43 | 0.20 | 0.66 |  | Exercise plus nutrition estimate. | Exercise+nutrition synthesis retained as exercise-based adjunct evidence; nutrition-only and electroacupuncture syntheses excluded from the exercise-based pool. |
| Zhuang 2022 | Appendicular skeletal muscle mass index | Resistance training | No physical activity control | SMD | 0.72 | 0.24 | 1.21 | 0% |  |  |
| Zhuang 2022 | Appendicular skeletal muscle mass index | Mixed / combined training | No physical activity control | SMD | 0.70 | 0.22 | 1.19 | 0% |  |  |
| Qiu 2025 | Fat-free mass | Multicomponent training (MCT) | Control | MD | 5.21 | 1.51 | 8.91 |  | NMA estimate. |  |
| Chen 2025 | Weight | Any exercise | Control | MD | -3.45 | -5.61 | -1.30 |  |  |  |
| Chen 2025 | Weight | Resistance training | Control | MD | -0.61 | -4.06 | 2.84 |  |  |  |
| Chen 2025 | Weight | Aerobic training | Control | MD | -6.07 | -9.89 | -2.25 |  |  |  |
| Chen 2025 | Weight | Mixed training | Control | MD | -4.40 | -8.40 | -0.40 |  |  |  |
| Hita-Contreras 2018 | BMI | EXE alone | Control | MD | -0.15 | -0.65 | 0.35 | 0% |  | Exercise-alone and exercise+supplement syntheses retained as exercise-based evidence. |
| Hita-Contreras 2018 | Standardized skeletal muscle mass index | EXE alone + EXE+SUPPL | Control | SMD | 0.40 | 0.03 | 0.76 | 0% | Secondary pooled estimate reported as standardized skeletal muscle mass index. | Exercise-alone and exercise+supplement syntheses retained as exercise-based evidence. |
| Hita-Contreras 2018 | Total fat mass | EXE alone + EXE+SUPPL | Control | MD | -1.77 | -2.49 | -1.04 | 21% |  | Exercise-alone and exercise+supplement syntheses retained as exercise-based evidence. |
| Hita-Contreras 2018 | Trunk fat mass | EXE alone + EXE+SUPPL | Control | MD | -0.82 | -1.22 | -0.42 | 16% |  | Exercise-alone and exercise+supplement syntheses retained as exercise-based evidence. |
| Hita-Contreras 2018 | Waist circumference | EXE alone + EXE+SUPPL | Control | MD | -1.40 | -1.99 | -0.81 | 12% |  | Exercise-alone and exercise+supplement syntheses retained as exercise-based evidence. |
| Hita-Contreras 2018 | Weight | EXE alone | Control | MD | -0.29 | -1.44 | 0.86 | 0% |  | Exercise-alone and exercise+supplement syntheses retained as exercise-based evidence. |

Displayed estimates are published pooled or network estimates from the included reviews. They were not statistically re-pooled across reviews.

# **Table S10. Review-level pooled effects for primary physical-function outcomes.**

| **Review** | **Outcome** | **Intervention** | **Comparator** | **Metric** | **Effect** | **CI low** | **CI high** | **I2** | **Notes** | **Exercise-based pool note** |
| --- | --- | --- | --- | --- | --- | --- | --- | --- | --- | --- |
| Chen 2025 | Walking speed (paper label WS) | Any exercise | Control | MD | 0.20 | 0.07 | 0.33 | 86% |  |  |
| Hita-Contreras 2018 | Gait speed | EXE alone + EXE+SUPPL | Control | MD | 0.05 | 0.03 | 0.07 | 0% |  | Exercise-alone and exercise+supplement syntheses retained as exercise-based evidence. |
| Qiu 2025 | Gait speed | Multicomponent training (MCT) | Control | MD | 0.35 | 0.30 | 0.41 |  |  |  |
| Qiu 2025 | Gait speed | Combined resistance + aerobic training (CT) | Control | MD | 0.14 | 0.06 | 0.21 |  |  |  |
| Wei 2025 | Gait speed | Any exercise | Control | MD | 0.88 | 0.65 | 1.11 |  |  |  |
| Wei 2025 | Gait speed | Resistance training (RT) | Control | MD | 0.96 | 0.62 | 1.30 |  |  |  |
| Wei 2025 | Gait speed | Combined exercise (CE) | Control | MD | 0.81 | 0.49 | 1.12 |  |  |  |
| Yin 2020 | Gait speed | Exercise-based intervention | Usual care / blank control | MD | 0.13 | 0.08 | 0.18 | 0% |  | Exercise-based and exercise+nutrition syntheses retained; nutrition-only and electroacupuncture syntheses excluded from the exercise-based pool. |
| Zhuang 2022 | Gait speed | Mixed / combined training | No physical activity control | SMD | 0.71 | 0.23 | 1.18 | 37% |  |  |
| Chen 2025 | Grip strength (paper label GS) | Any exercise | Control | MD | 2.02 | 0.02 | 4.01 | 51% | Paper uses GS for grip strength and WS for walking speed; harmonized here accordingly. |  |
| Hita-Contreras 2018 | Grip strength | EXE alone + EXE+SUPPL | Control | MD | 1.30 | 0.58 | 2.01 | 20% |  | Exercise-alone and exercise+supplement syntheses retained as exercise-based evidence. |
| Qiu 2025 | Handgrip strength | Multicomponent training (MCT) | Control | SMD | 0.87 | 0.19 | 1.50 |  |  |  |
| Qiu 2025 | Handgrip strength | Resistance training (RT) | Control | SMD | 0.84 | 0.43 | 1.25 |  | RT ranked best by SUCRA for HGS. |  |
| Wei 2025 | Grip strength | Any exercise | Control | MD | 2.82 | 2.05 | 3.59 |  |  |  |
| Wei 2025 | Grip strength | Resistance training (RT) | Control | MD | 3.43 | 2.03 | 4.84 |  |  |  |
| Wei 2025 | Grip strength | Combined exercise (CE) | Control | MD | 2.64 | 1.71 | 3.57 |  |  |  |
| Yin 2020 | Grip strength | Exercise-based intervention | Usual care / blank control | MD | 1.63 | 0.94 | 2.32 |  | I2 not stated in extracted source details for this pooled result. | Exercise-based and exercise+nutrition syntheses retained; nutrition-only and electroacupuncture syntheses excluded from the exercise-based pool. |
| Zhuang 2022 | Handgrip strength | Resistance training | No physical activity control | SMD | 1.06 | 0.22 | 1.91 | 90% |  |  |
| Chen 2025 | Knee extension strength | Any exercise | Control | MD | 2.14 | 0.47 | 3.82 | 19% |  |  |
| Chen 2025 | Knee extension strength | Resistance training | Control | MD | 4.85 | 1.97 | 7.72 |  |  |  |
| Chen 2025 | Knee extension strength | Aerobic training | Control | MD | -0.08 | -2.88 | 2.73 |  | Value from results section; not in abstract. |  |
| Chen 2025 | Knee extension strength | Mixed training | Control | MD | 1.73 | -1.31 | 4.78 |  |  |  |
| Guo 2025 | Timed up-and-go | Resistance training | Control | WMD | -2.23 | -2.96 | -1.49 |  | Lower time was favorable. |  |
| Guo 2025 | Timed chair-rise | Resistance training | Control | WMD | 5.20 | 3.98 | 6.43 |  | Higher repetitions were favorable. |  |

Displayed estimates are published pooled or network estimates from the included reviews. They were not statistically re-pooled across reviews.

# **Table S11. Review-level pooled effects for secondary metabolic and inflammatory outcomes.**

| **Review** | **Outcome** | **Intervention** | **Comparator** | **Metric** | **Effect** | **CI low** | **CI high** | **I2** | **Notes** | **Exercise-based pool note** |
| --- | --- | --- | --- | --- | --- | --- | --- | --- | --- | --- |
| Chen 2025 | IGF-1 | Any exercise | Control | MD | 0.59 | 0.24 | 0.95 |  |  |  |
| Chen 2025 | IGF-1 | Resistance training | Control | MD | 0.79 | 0.05 | 1.52 |  |  |  |
| Chen 2025 | IGF-1 | Mixed training | Control | MD | 1.01 | 0.45 | 1.56 |  |  |  |
| Chen 2025 | IL-6 | Any exercise | Control | MD | -0.01 | -0.27 | 0.24 |  |  |  |
| Polo-Ferrero 2025 | Biomarker composite | Resistance training | No intervention | SMD | 0.10 | -0.28 | 0.49 | 0% | Composite biomarker meta-analysis including LDL/HDL/TG/CRP-related measures. |  |
| Wei 2025 | CRP | Exercise group pre-post | Within-group pre-post | MD | -0.06 | -0.38 | 0.27 |  | Within-group pre-post analysis only. |  |
| Wei 2025 | Glucose | Exercise group pre-post | Within-group pre-post | MD | -0.12 | -0.59 | 0.35 |  | Not a between-group contrast; within-group pre-post analysis only. |  |
| Wei 2025 | HDL-C | Exercise group pre-post | Within-group pre-post | MD | 0.20 | -0.11 | 0.51 |  | Within-group pre-post analysis only. |  |
| Wei 2025 | IL-6 | Exercise group pre-post | Within-group pre-post | MD | -0.15 | -0.46 | 0.17 |  | Within-group pre-post analysis only. |  |
| Wei 2025 | IL-6 | Combined exercise (CE) pre-post | Within-group pre-post | MD | -0.51 | -1.07 | 0.06 |  | Within-group pre-post CE subgroup. |  |
| Wei 2025 | Insulin | Exercise group pre-post | Within-group pre-post | MD | -0.93 | -2.10 | 0.24 |  | Within-group pre-post analysis only. |  |
| Wei 2025 | Insulin | Combined exercise (CE) pre-post | Within-group pre-post | MD | -1.73 | -3.21 | -0.25 |  | Within-group pre-post CE subgroup. |  |
| Wei 2025 | LDL-C | Exercise group pre-post | Within-group pre-post | MD | 0.05 | -0.26 | 0.35 |  | Within-group pre-post analysis only. |  |
| Wei 2025 | TNF-alpha | Exercise group pre-post | Within-group pre-post | MD | -0.14 | -0.42 | 0.14 |  | Within-group pre-post analysis only. |  |
| Wei 2025 | Total cholesterol | Exercise group pre-post | Within-group pre-post | MD | -0.38 | -0.71 | -0.06 |  | Within-group pre-post analysis only. |  |
| Wei 2025 | Triglycerides | Exercise group pre-post | Within-group pre-post | MD | -1.58 | -7.22 | 4.06 |  | Within-group pre-post analysis only. |  |
| Zhuang 2022 | IGF-1 | Any physical activity | No physical activity control | SMD | 0.38 | 0.11 | 0.66 | 0% |  |  |

Displayed estimates are published pooled or network estimates from the included reviews. They were not statistically re-pooled across reviews. Within-group pre-post estimates were treated as exploratory and were not interpreted as between-group treatment effects.

# **Supplementary Figure S1**

Figure S1. Pairwise Jaccard overlap heatmap for the exercise-based strict main pool. The heatmap displays pairwise Jaccard overlap indices among the eight included reviews after primary-study/unit-level reconciliation.


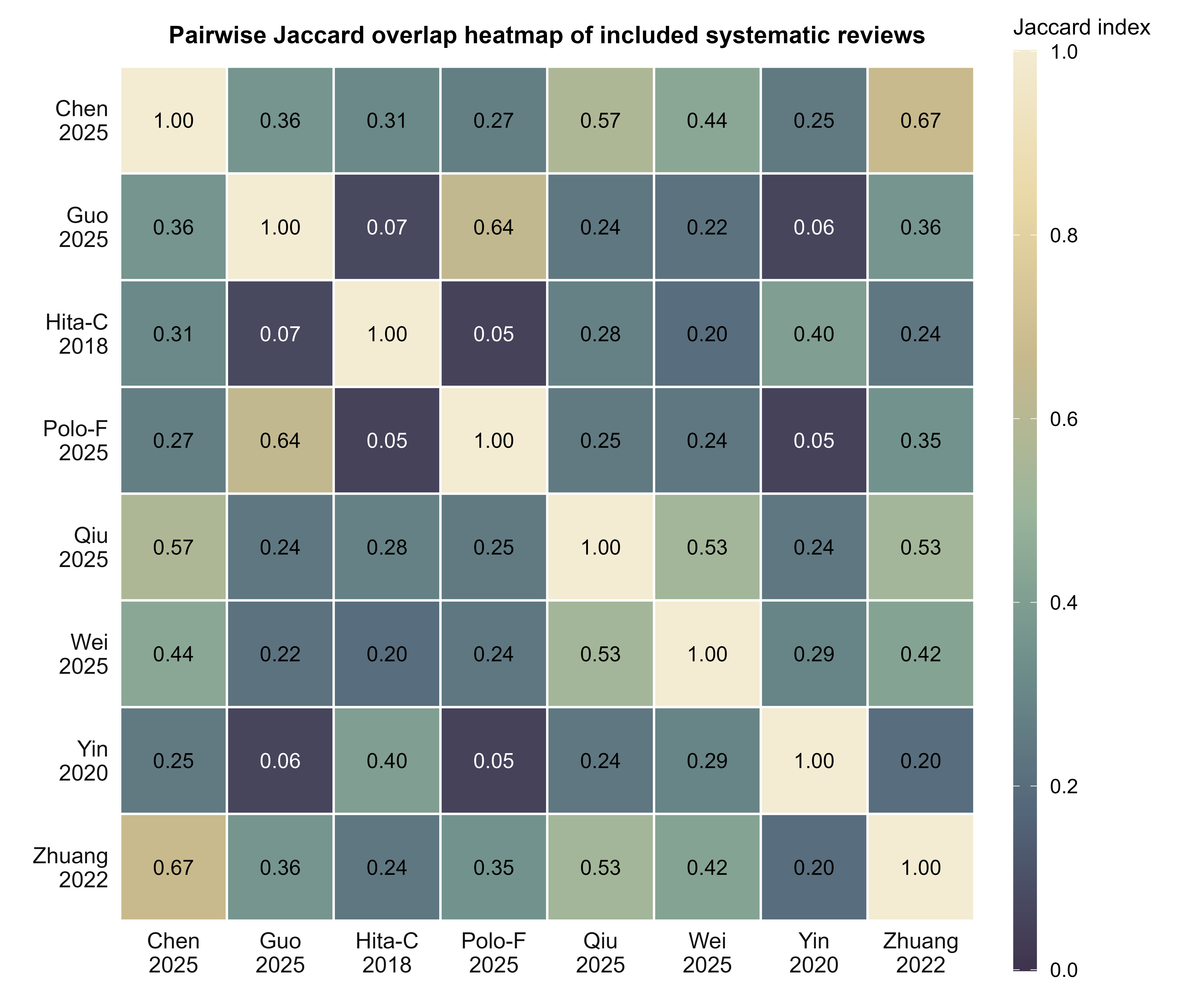

Supplement: Supplementary file 1 [file Table_1.DOCX]
