# Supplementary material for: Exercise-based interventions for sarcopenic obesity in middle-aged and older adults: an umbrella review of systematic reviews with pairwise meta-analyses and network meta-analyses
Source: Front Nutr. 2026 Jun 10;13:1859967. doi: 10.3389/fnut.2026.1859967 (PMC13291741; doi:10.3389/fnut.2026.1859967)
Supplement: Supplementary file 2 [file Table_2.DOCX]

**PRISMA 2020 Checklist**

Manuscript: Exercise-based interventions for sarcopenic obesity in middle-aged and older adults: an umbrella review of systematic reviews with pairwise meta-analyses and network meta-analyses

Locations refer to the rendered revised manuscript with continuous line numbering enabled and to Supplementary File 1 where applicable.

| **Section** | **Item** | **Checklist item** | **Location** | **Notes** |
| --- | --- | --- | --- | --- |
| Title | 1 | Identify the report as a systematic review. | Title, lines 2–4 | Umbrella review of systematic reviews is stated in the title. |
| Abstract | 2 | Provide a structured summary including objectives, methods, results and interpretation. | Abstract, lines 14–44 | Structured Introduction, Methods, Results, Discussion and registration statement. |
| Introduction | 3 | Describe the rationale for the review in the context of existing knowledge. | Introduction, lines 47–93 | Includes SO burden, definition heterogeneity, prior umbrella review and rationale. |
| Introduction | 4 | Provide an explicit statement of the objective or question. | Introduction, lines 78–87 | Objectives are stated at the end of the Introduction. |
| Methods | 5 | Specify eligibility criteria for studies and reports. | Methods, lines 103–134 | Includes population, intervention, comparator, outcomes, study design and exclusions. |
| Methods | 6 | Specify all databases, registers, websites, organizations and other sources searched. | Methods, lines 135–145; Supplementary Table S1 | Six databases and reference-list checking are reported. |
| Methods | 7 | Present the full search strategies for all databases and sources. | Supplementary Table S1 | Full electronic strategies and per-database yields are provided. |
| Methods | 8 | Specify the selection process, including number of reviewers and resolution of disagreements. | Methods, lines 146–158 | Independent screening and adjudication are described. |
| Methods | 9 | Specify the data collection process, including number of reviewers. | Methods, lines 159–169 | Independent extraction and verification are described. |
| Methods | 10a | List and define all outcomes for which data were sought. | Methods, lines 111–118 and 160–166 | Body composition, physical function, metabolic and inflammatory outcomes are defined. |
| Methods | 10b | List and define other variables for which data were sought. | Methods, lines 159–169; Supplementary Table S2 | Review design, population, SO definition, age scope, intervention scope, search end date and other variables reported. |
| Methods | 11 | Specify methods used to assess risk of bias or methodological quality. | Methods, lines 170–183; Table 2; Supplementary Table S5 | AMSTAR-2 was used for review-level methodological quality. |
| Methods | 12 | Specify effect measures used for each outcome. | Methods, lines 216–222; Table 3; Supplementary Tables S9–S11 | MD, WMD, SMD and network estimates reported as extracted. |
| Methods | 13a | Describe processes used to decide which studies were eligible for each synthesis. | Methods, lines 119–131; Supplementary Table S3 | Exercise-based retention rule and partial extraction rules are reported. |
| Methods | 13b | Describe methods required to prepare data for synthesis. | Methods, lines 159–169 and 184–193 | Review-level extraction and overlap-matrix harmonization are described. |
| Methods | 13c | Describe methods used to tabulate or visually display results. | Methods, lines 194–208 and 216–235; Figure 2; Table 3; Supplementary Tables S9–S11 | Descriptive evidence map and tabulation approach are described. |
| Methods | 13d | Describe methods used to synthesize results. | Methods, lines 216–235 | Review-level estimates were not statistically re-pooled because overlap was very high. |
| Methods | 13e | Describe methods used to explore heterogeneity. | Methods, lines 194–208 and 216–235; Supplementary Tables S9–S11 | Heterogeneity statistics were extracted where available and used descriptively. |
| Methods | 13f | Describe sensitivity analyses, if any. | Methods, lines 216–235; Results, lines 332–341 | Sensitivity summaries are descriptive and not interpreted as independent statistical tests. |
| Methods | 14 | Describe methods used to assess reporting bias. | Methods, lines 227–230 | Formal publication-bias testing was not performed because reviews were few, measures differed and overlap was extensive. |
| Methods | 15 | Describe methods used to assess certainty or confidence in the body of evidence. | Methods, lines 209–215; Table 3 | Low/very-low umbrella-level certainty qualifiers were used; no formal de novo GRADE/JBI/CINeMA assessment was performed. |
| Results | 16a | Describe results of search and selection process. | Results, lines 237–248; Figure 1 | PRISMA counts are reported. |
| Results | 16b | Cite studies that appear to meet criteria but were excluded and explain why. | Results, lines 237–248; Supplementary Table S4 | Citation-level full-text exclusions and reasons are provided. |
| Results | 17 | Cite each included study and present its characteristics. | Results, lines 249–265; Table 1; Supplementary Table S2 | Eight included reviews and characteristics are presented. |
| Results | 18 | Present risk-of-bias or methodological-quality assessments. | Results, lines 266–275; Table 2; Supplementary Table S5 | AMSTAR-2 summary and complete item-level matrix are provided. |
| Results | 19 | Present results of individual studies or source reviews. | Table 3; Supplementary Tables S9–S11 | Representative and detailed review-level estimates are tabulated. |
| Results | 20a | For each synthesis, briefly summarize characteristics and contributing studies. | Results, lines 249–265 and 284–331 | Outcome-domain sections summarize contributing reviews. |
| Results | 20b | Present results of all syntheses conducted. | Results, lines 284–331; Table 3; Figure 2 | Outcome-domain synthesis results are reported. |
| Results | 20c | Present results of investigations of heterogeneity. | Table 3; Supplementary Tables S9–S11 | I² values are reported where available; synthesis is descriptive. |
| Results | 20d | Present results of sensitivity analyses. | Results, lines 332–341 | Descriptive sensitivity findings are summarized. |
| Results | 21 | Present assessments of reporting bias. | Methods, lines 227–230 | Formal reporting-bias testing was not conducted for stated methodological reasons. |
| Results | 22 | Present assessments of certainty or confidence in evidence. | Abstract, lines 31–32; Table 3; Discussion, lines 351–356; Limitations, lines 488–500 | Low/very-low umbrella-level certainty is reported. |
| Discussion | 23a | Provide a general interpretation of results. | Discussion, lines 342–356; Conclusion, lines 502–511 | Findings are interpreted cautiously. |
| Discussion | 23b | Discuss limitations of the evidence. | Discussion, lines 442–460; Limitations, lines 488–500 | Overlap, AMSTAR-2 confidence, heterogeneity and missing safety/adherence data are discussed. |
| Discussion | 23c | Discuss limitations of the review process. | Limitations, lines 488–500 | Narrow main pool, no primary-study meta-analysis and no formal GRADE/JBI/CINeMA are acknowledged. |
| Discussion | 23d | Discuss implications for practice, policy and future research. | Clinical and research implications, lines 461–481 | Practice and future-research implications are stated cautiously. |
| Other information | 24a | Provide registration information. | Abstract, lines 43–44; Methods, lines 96–98 | PROSPERO number and URL are provided. |
| Other information | 24b | Indicate where the review protocol can be accessed. | Abstract, lines 43–44; Methods, lines 96–98 | PROSPERO URL provides access to the registration record. |
| Other information | 24c | Describe and explain amendments to the protocol. | Not applicable/not reported | No protocol amendments are reported in the manuscript. |
| Other information | 25 | Describe sources of financial or non-financial support. | Funding, lines 543–545 | No financial support was received. |
| Other information | 26 | Declare competing interests. | Conflict of interest, lines 546–548 | No commercial or financial conflicts are declared. |
| Other information | 27 | Report availability of data, code and other materials. | Data availability statement, lines 522–528 | Datasets and supplementary materials are described. |
